# Binary and ternary complexes of epinephrine with alginate and biologically and environmentally relevant metal cations

**DOI:** 10.3389/fchem.2023.1189308

**Published:** 2023-04-25

**Authors:** Anna Irto, Rosalia Maria Cigala, Chiara Alessandrello, Concetta De Stefano, Giuseppe Gattuso, Francesco Crea

**Affiliations:** Dipartimento di Scienze Chimiche, Biologiche, Farmaceutiche ed Ambientali, Messina, Italy

**Keywords:** epinephrine, alginate, Cu^2+^ and UO_2_^2+^ sequestration, ligand-ligand interaction, ternary complexes, extra-stability, DOSY NMR

## Abstract

The speciation of epinephrine (*Eph* ^−^) in the presence of alginate (*Alg* ^2-^) and two biological and environmental relevant metal cations (Cu^2+^, UO_2_^2+^) was investigated at *T* = 298.15K, *I* = 0.15–1.00 mol dm^−3^ in NaCl_(aq)_. The formation of binary and ternary complexes was evaluated and, since epinephrine can behave as a zwitterion, the *Eph* ^−^/*Alg* ^2-^ interaction was studied by means of DOSY NMR. The dependence of the equilibrium constants on ionic strength was studied using an extended Debye-Hückel type equation and the SIT approach. The effect of temperature was investigated by means of isoperibolic titration calorimetry: the entropic contribution was the driving force for the Cu^2+^/*Eph* ^−^ complexes formation. The sequestering ability of *Eph* ^−^ and *Alg* ^2-^ on Cu^2+^, evaluated by the pL_0.5_ calculation, increased with pH and ionic strength. The determination of pM parameter showed that *Eph* ^−^ had a higher Cu^2+^ affinity with respect to *Alg* ^2-^. The formation of *Eph* ^−^/*Alg* ^2-^ species was also investigated by UV-Vis spectrophotometry and ^1^H NMR measurements. The ternary Cu^2+^/*Eph* ^−^/*Alg* ^*2-*^ and Cu^2+^/UO_2_^2+^/*Eph* ^−^ interactions were also studied. The “extra-stability” calculated for the mixed ternary species confirmed that their formation was thermodynamically favorable.

## 1 Introduction

The onset of more frequent disorders owing to the accumulation of metal cations in the human body or in natural fluids is encouraging the scientific community to look at new chelators (Raymond and Carrano, 1979; Schiewer and Volesky, 1996; Schneider and Rubio, 1999; Barbero et al., 2013; Berto et al., 2018; Irto et al., 2018; Santander et al., 2021), which alone, or in combined effect together with other ligands, could efficiently sequester metal cations without involving drawbacks such as toxicity, economic inaccessibility, and absence of affinity towards biological membranes (Irto et al., 2021).

In recent decades, many molecules (such as amino acids, hormones, and carboxylic acids) participating in normal human body functions or compounds extracted by natural matrices (Grgas-Kužnar et al., 1974; Gergely et al., 1981; Kiss and Gergely, 1983; Schiewer and Volesky, 1996; Gerard and Hanane, 1997; Schneider and Rubio, 1999; Davis et al., 2003; Cigala et al., 2015; Piperea-Şianu et al., 2015; Idota et al., 2016; Vione et al., 2016) have been tested as potential metal chelators for biological or environmental applications.

**FIGURE 1:**
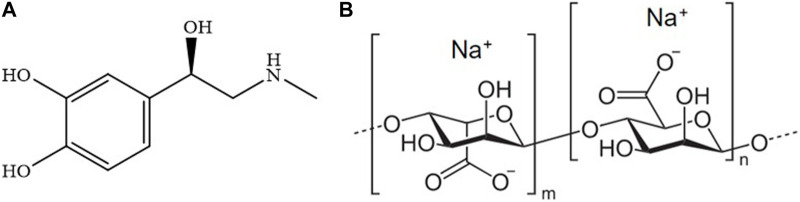
Molecular structures of epinephrine **(A)** and sodium alginate **(B)**.

Among them, epinephrine (Figure 1A), also known as adrenaline, is a pharmacologically active substance belonging to the catecholamine family. Its structure features an aromatic ring substituted with two -OH in *ortho* positions and an alkyl side chain with hydroxyl and secondary amino groups as substituents. 

When the human body is in a situation of severe stress, the medullary adrenal gland, stimulated by the autonomic nervous system, releases epinephrine into the bloodstream in a three-step process. In particular, the hypothalamus produces hormones stimulating the pituitary gland. This latter in turn makes corticotrophin hormones that encourage the adrenal glands for the production of corticosteroid hormones and neurotransmitters like adrenaline (Barrington, 2000). The main functions of epinephrine are: increasing heart rate, the facilitation of blood flow to the muscles and brain, the relaxation of smooth muscle, help with the conversion of glycogen to glucose in the liver, raising the level of sugar in the blood, the acceleration of breathing, the modulation of memory consolidation, and employment as a haemostatic agent to prolong the action of local anaesthetics in postoperative treatments (Cahill and Alkire, 2003; Flint et al., 2007). Furthermore, it is used in the medical field for the treatment of glaucoma, asthma, and cardiac arrest.

On the other hand, among natural organic substances in the aquatic ecosystems, many biopolymers, such as cellulose, lignin, chitin and pectin derivatives, alginates, and humic substances are known to strongly sequester metal cations for possible applications in wastewater treatment and heavy metals removal from contaminated sites (Pandey et al., 2000; Santander et al., 2021), and also as possible carriers of molecules of biological interest.

Alginate (Figure 1B) is a polysaccharide derived from alginic acid, a copolymer of β-D-mannuronic and α-L-guluronic acids (Nussinovitch, 1997; Lee and Mooney, 2012) residues linked to each other by means of a 1,4 bond (De Stefano et al., 2005). It is mainly extracted from brown seaweed, such as the *L. hyperborea* in the North Atlantic coastal regions, the *A. nodosum* from North Europe and Canada, and the *M. pyrifera* from the western coasts of America (Nussinovitch, 1997). Alginate is usually found in the sodium(I), calcium(II), or magnesium(II) form: the presence of these metal cations makes it more stable than alginic acid, especially when alginate is interacting with bivalent metals (Pereira and Cotas, 2019).

Features such as high viscosity, gelling properties, and high stability make alginate an important industrial polysaccharide. Alginate has several applications in the pharmaceutical industry such as for the formation of gels, as stabilizing agents, and for localized drug delivery. The use of alginate hydrogels for tissue drug delivery is widely used nowadays, and it has often been encapsulated with several drugs to enhance its wound healing properties.

It is used as a 3D culture matrix because it provides support for the integration of cells and acts as a platform for cellular growth.

Unfortunately, the data reported in the literature on the stability of metal/alginate complexes are not always homogeneous and sometimes not in agreement with each other. These discrepancies could be possibly due to the different experimental conditions (such as pH range, ionic strengths, supporting electrolyte, and metal-to-ligand concentration ratios) used by the authors, or to the electrostatic effects owing to the nature and molecular weight of the polyelectrolyte and to the various thermodynamic models used to describe the alginate acid-base behavior (Mohammed et al., 2022).

The knowledge of a ligand’s thermodynamic properties (equilibrium constants, parameters for the dependence on *I*/mol dm^−3^, and temperature *T*/K) and of the different chemical forms in which it is distributed, namely the speciation (Templeton et al., 2000), at experimental conditions simulating those of real multicomponent systems (biological, natural fluids), are very important tools for gaining information about ligand bioavailability, toxicity, and environmental impact and for possible applications in real biological, environmental, and pharmaceutical cases studies.

In this light, this contribution reports the results obtained by performing a multi-technique (ISE-[H^+^] potentiometry, UV-Vis spectrophotometry, ^1^H NMR, isoperibolic calorimetry, and thermogravimetry) speciation study on the binary and ternary interactions of epinephrine (*Eph* ^−^) with alginate (*Alg* ^2-^) and two metal cations of biological and environmental relevance, such as Cu^2+^ and UO_2_^2+^. Since adrenaline can behave as a zwitterion in aqueous solution (Antikainen and Witikainen, 1973), the possible ligand-ligand interaction between *Eph* ^−^ and *Alg* ^2-^ was also studied by ISE-[H^+^] potentiometry, UV-Vis spectrophotometry, and ^1^H NMR.

The investigations were carried out at *T* = 298.15K in NaCl aqueous solution, the main inorganic component of several biological (Buffle, 1988; Millero, 2001) and natural (Lentner, 1981) fluids, and at different ionic strengths. The dependence on ionic strength of the equilibrium constants of the Cu^2+^/*Eph* ^−^ and Cu^2+^/*Alg* ^2-^ species was studied using an extended Debye-Hückel type equation and, for the first system, also with the Specific ion Interaction Theory (SIT) approach (Biederman, 1975; Biederman, 1986; Irto et al., 2019a). The effect of temperature on Cu^2+^/*Eph* ^−^ speciation was investigated by isoperibolic titration calorimetry, allowing the determination of enthalpy changes values for the formation of some complexes. Thermogravimetry (TGA) measurements were carried out on solid samples collected at the end of potentiometric titrations to determine the precipitate stoichiometry and gaining information on the thermal stability of the systems.

The Cu^2+^ sequestering ability and affinity of epinephrine and alginate were evaluated by the determination of the pL_0.5_ (Crea et al., 2014) and pM (Raymond and Carrano, 1979) parameters at different ionic strength and pH conditions.

The ternary Cu^2+^/*Eph* ^−^/*Alg* ^*2-*^ and Cu^2+^/UO_2_^2+^/*Eph* ^−^ interactions were also investigated since the knowledge of the possible formation of mixed species is fundamental for the treatment of many real biological and environmental problems and because in many cases the formation of these mixed species increases the solubility and the availability of the metal cations. Furthermore, the extra-stability (Beck and Nagypál, 1991) of selected ternary complexes was determined to evaluate if the formation of mixed species could be thermodynamically favored with respect to the corresponding polynuclear binary complexes.

## 2 Materials and methods

### 2.1 Chemicals

Sodium hydroxide and hydrochloric acid solutions were prepared using Riedel-de Häen (Seelze, Germany) concentrated ampoules and standardized by means of potassium hydrogen phthalate and sodium carbonate, respectively. The base solutions were preserved from atmospheric CO_2_ effects by employing soda lime traps. Fresh adrenaline solutions were prepared by weighing the (-)-epinephrine solid product, purchased by Sigma-Aldrich (Milan, Italy), without any purification. The ligand purity was tested by potentiometry by means of alkalimetric titrations and the results showed it to be >99%. Alginic acid sodium salt from *brown algae* (Sigma-Aldrich, Milan, Italy) was weighed for the preparation of alginate solutions. CuCl_2_·2H_2_O and Cu(NO_3_)_2_ hydrate salts, both purchased by Fluka (Darmstadt, Germany), were used to prepare the metal solutions. They were standardized employing EDTA standard solutions (Flaschka, 1959) and their purity was always ≥98%. UO_2_^2+^ nitrate and diacetate salts (Fluka, Darmstadt, Germany) were used. The gravimetric determination of uranium after ignition to the U_3_O_8_ oxide was performed for the determination of uranyl products purity (Crea et al., 2003). The preparation of ionic medium solutions was carried out by weighing the pure sodium chloride salt purchased by Fluka (Darmstadt, Germany). The NaCl solid product was formerly dried in an oven for 2 hours at *T* = 383.15K. All the solutions were prepared by employing analytical grade water (R = 18 MΩ cm^−1^), reagents of the highest available purity, and grade A glassware.

### 2.2 Apparatuses and procedures

#### 2.2.1 Potentiometric titrations

Potentiometric measurements were carried out using a Metrohm (Herisau, Switzerland) Titrando 809 model and a potentiometer with a Ross type 8102 combined glass electrode (Thermo-Orion, Waltham, MA United States) plugged to an automatic burette. The mentioned apparatus was connected to a PC and automatic titrations were performed employing a Metrohm TiAMO 2.5 software for checking for titrant delivery, e.m.f. stability and data acquisition. The estimated accuracy values for e.m.f. (electromotive force) and titrant volume readings were ±0.15 mV and ±0.003 cm^3^, respectively. The experiments were carried out in thermostated cells under magnetic stirring. Purified presaturated N_2(g)_ was bubbled into the measurements solutions to exclude O_2(g)_ and CO_2(g)_ presence inside.

The potentiometric measurements were carried out at *T* = 298.15K, at different metals and ligands concentrations, metal:ligand molar ratios, and ionic strengths, as reported in Table 1.

**TABLE 1:** Experimental details of the potentiometric investigations performed at *T* = 298.15K in NaCl_(aq)_.

System	*I*/mol dm^−3^	*c*_Cu_^2+^ ^a^	*c*_UO2_^2+^ ^a^	*c*_*Eph*_^*-*^ ^a^	*c*_*Alg*_^2-^ ^a^	pH range
H^+^/*Alg* ^*2-*^	0.15–1.00	-	-	-	0.60–1.30	2.0–10.5
Cu^2+^/*Alg* ^*2-*^	0.15–1.00	0.60–1.00	-	-	0.70–2.20	2.5–10.5
Cu^2+^/*Eph* ^*-*^	0.15–1.00	1.00–2.00	-	1.00–4.00	-	3.0–8.5
*Eph* ^−^/*Alg* ^*2-*^	0.15	-	-	1.50–4.50	1.50–3.00	2.0–10.0
Cu^2+^/*Eph* ^−^/*Alg* ^*2-*^	0.15	1.05	-	1.00–3.60	1.00–3.00	2.0–10.0
Cu^2+^/UO_2_^2+^/*Eph* ^-^	0.16	5.00–10.00	5.00–10.00	8.00	-	2.0–8.5

^a^ in mmol dm^−3^.

In the case of the H^+^/*Alg* ^*2-*^ *,* Cu^2+^/*Alg* ^*2-*^ and Cu^2+^/*Eph* ^−^/*Alg* ^*2-*^ systems, acidimetric titrations were performed using titrant solutions of standard hydrochloric acid. On the contrary, for the other systems, alkalimetric measurements with standard sodium hydroxide solutions were carried out.

#### 2.2.2 Spectrophotometric measurements

Spectrophotometric experiments were carried out by means of a Varian (Agilent Scientific Instruments, CA, United States) Cary 50 UV–Vis spectrophotometer, equipped with an optic fiber probe (path length: 1 cm). A personal computer was connected to the instrument and Varian Cary WinUV software was used for the acquisition of absorbance (A) signal vs. wavelength (λ/nm). Concurrently, potentiometric data were recorded by means of a combined Ross type 8102 glass electrode (Thermo-Orion, Waltham, MA United States) connected to a potentiometer. The titrant solutions were delivered in the thermostated experiments cells, employing an automatic burette, Metrohm (Herisau, Switzerland) 665 model. The solution’s homogeneity was ensured using a stirring bar. To rule out the presence of oxygen and carbon dioxide from the measurement solutions, before starting the experiments, gaseous nitrogen was bubbled through for 5 min.

The binding ability of epinephrine towards Cu^2+^ was studied by titrating with standard sodium hydroxide 25 cm^3^ solutions containing the ligand (*c*_*Eph*_^−^ = 0.05–0.10 mmol dm^−3^), the metal cation (*c*_Cu_^2+^ = 0.05–0.10 mmol dm^−3^), hydrochloric acid (*c*_H_^+^ = 5.00–8.00 mmol dm^−3^), and NaCl at *I* = 0.15 mol dm^−3^, in the pH range 3.0–11.0 and 200 ≤ λ/nm ≤ 450.

UV-Vis investigations into the *Eph* ^−^/*Alg* ^2-^ system were performed in the absence of ionic medium at *T* = 298.15K and in the wavelength range 200 ≤ λ/nm ≤ 450. In these investigations, 25 cm^3^ of solutions containing epinephrine, alginate, or both the ligands at *c*_*Eph*_^*-*^ = *c*_*Alg*_^*2*-^ = 0.12 mmol dm^−3^ were titrated with standard HCl (0.0985 mmol dm^−3^) or NaOH (0.0956 mmol dm^−3^) solutions in the pH ranges 8.7–2.0 and 8.5–10.5, respectively.

#### 2.2.3 Calorimetric titrations

The study of the heat of the reactions involved in the Cu^2+^/*Eph* ^*-*^ complexation was performed using a Calorimetry Sciences Corporation (CSC, Utah, United States) calorimeter (Model 4285) equipped with a constant temperature bath (Mod. 7211). The experiments were performed by titrating a 25 cm^3^ solution of ligand (*c*_*Eph*_^*-*^ = 2.5–5 mmol dm^−3^), previously salified with sodium hydroxide, with a titrant solution of Cu(NO_3_)_2_ hydrate at *c*_Cu_^2+^ = 0.0715 mol dm^−3^ in NaCl_(aq)_ at *I* = 0.50 mol dm^−3^, delivered by means of a Hamilton syringe, model 1002TLL (Sigma Aldrich, Milan, Italy), with a 2.5 cm^3^ capacity. The pH range investigated was 10.0 ≥ pH ≥ 4.5. Measurements were repeated at least three times for each selected experimental condition. The dilution enthalpy was measured before each experiment. The accuracies of calorimetric apparatus and of titrant volume were ±0.008 J and 0.001 cm^3^, respectively. Calibration measurements were carried out by titrating a THAM (tris-(hydroxymethyl)amino-methane) buffer with hydrochloric acid. The enthalpy change values to be used in the calculations for the water ionization has been already reported in the literature (De Stefano et al., 2001).

#### 2.2.4 Thermogravimetric measurements

A Perkin Elmer Pyris Diamond thermobalance was employed for the thermal analysis and the analytical data were elaborated using the version 2.6 Muse Measurement thermal analysis software (supplied by Perkin Elmer Corp.). Precipitate collected at the end of Cu^2+^/*Eph* ^−^ potentiometric measurements were filtered with 0.45 µm cellulose filters. The solids were washed with little amounts of ultrapure water and treated with small aliquots of acetone and dried under vacuum. Approximately 2–10 mg of the obtained samples was heated in platinum crucibles at the following conditions, allowing for the best possible resolution of the thermogravimetric curves: a temperature range of 293.15 ≤ *T*/K ≤ 1123.15, an atmosphere of gaseous mixture of nitrogen and oxygen with 80% and 20% v/v, respectively, a flow rate of 100 cm^3^ min^−1^, and a scanning rate of 283.15 K min^−1^.

#### 2.2.5 ^1^H NMR measurements

NMR spectra were recorded at *T* = 298.15K in D_2_O on a Varian 500 MHz instrument equipped with a pulse-field gradient probe. 1,4-Dioxane (*δ*_H_ = 3.75 ppm) was used as an internal standard. ^1^H NMR spectra were recorded using solvent suppression pulse sequences (PRESAT). Diffusion-ordered NMR spectroscopy (DOSY) studies were performed using a Doneshot pulse sequence (Pelta et al., 2002), optimizing the experimental parameters according to the sample under investigation. Diffusion gradients were progressively incremented over 15 steps, varying the gradient strength from 1.8 to 50.0 gauss/cm. Sixteen transients were acquired for each increment, with a diffusion-gradient length of 2–4 ms and diffusion delays in the 50–300 m range.

For the experiments, solutions of epinephrine (*c*_*Eph*_^−^ = 2.29 mmol dm^−3^), alginate (*c*_*Alg*_^2-^ = 2.60 mmol dm^−3^), and *Eph* ^−^/*Alg* ^*2-*^ (*c*_*Eph*_^−^ = 2.29 mmol dm^−3,^ *c*_*Alg*_^2-^ = 2.60 mmol dm^−3^) were prepared in D_2_O without addition of NaCl. The pH of the solutions was ∼9.5.

### 2.3 Computer programs

The BSTAC computer program (De Stefano et al., 1997) was used for the determination of *E* ^0^, p*K*_*w*_, and *j*_*a*_ parameters, the reagents analytical concentration, and the equilibrium constants. UV-Vis spectrophotometric data were analyzed by employing HYPERQUAD 2008 (Gans et al., 1996). The least squares LIANA program (De Stefano et al., 1997) was used for the determination of Debye–Hückel and SIT parameters as well as for the calculation of the formation constants at infinite dilution. The elaboration of calorimetric data recorded by means of isoperibol titration calorimetry was performed through the ES5CM program (De Stefano et al., 1997). The calculations of the species formation percentages and the distribution diagrams were carried out using the HySS program (Alderighi et al., 1999).

### 2.4 Models for ionic strength dependence

An extended Debye-Hückel type equation (Eq. 1a) was employed to model the dependence on ionic strength of the stability constants of the Cu^2+^/*Eph* ^−^ and Cu^2+^/*Alg* ^*2-*^ species:

$$\log\beta_{pqr}=\log^{T}\beta_{pqr}–z^{*}\cdot DH+C\cdot I$$ (1a)

$$z^{*}=\sum {\left(charges\right)}_{reactants}^{2}-\sum {\left(charges\right)}_{products}^{2}$$ (1b)

where log^T^ *β*_pqr_ = equilibrium constant at infinite dilution:

The Debye- Hückel term (DH), can be expressed by:

$$DH=0.51\cdot \left(I^{0.5}/\left(1+1.5\cdot I^{0.5}\right)\right);$$

*C* = empirical parameter for the dependence of the formation constants on ionic strength.

Regarding the Cu^2+^/*Eph* ^−^ system, the equilibrium constants and ionic strengths were calculated on the molal (*m*, mol kg^−1^_H2O_) concentration scale and the Specific ion Interaction Theory (SIT) (Biederman, 1975; Biederman, 1986) equation was used. In this case, the *C* parameter of Eq. 1a is replaced by the ∆ε value (Eq. 2) and the interactions between opposite charge ions participating to the equilibria are considered for the calculations.

$$\Delta \varepsilon =\sum \varepsilon_{reactants}-\sum \varepsilon_{products}$$ (2)

For neutral species, the SIT coefficients are expressed by means of the Setschenow equation (Setschenow, 1889) and of the *k*_m_ parameter related to the activity coefficient by the Eq. 3

$$\log\gamma =k_{m}\cdot I$$ (3)

If all the interactions between the ionic components and species are considered, it is possible to calculate the single ε values of the Cu^2+^/*Eph* ^*-*^ ion pairs. In fact, the Δε parameter of Eq. 2 can be explicated to obtain the ion-pairs SIT coefficients for all the species involved in the equilibrium of formation of the complexes. If we consider each species of the Cu^2+^/*Eph* ^−^ system, the Δε can be explicated as (Eqs 4–8):

$$Cu(Eph{)}_{2\left(aq\right)}^{0};∆\varepsilon =\varepsilon \left({Cu}^{2+},{Cl}^{-}\right)+2\varepsilon \left(Eph^{-},{Na}^{+}\right)-k(Cu\left(Eph{)}_{2}^{0}\right)$$ (4)

$${Cu}_{2}(Eph{)}^{3+};∆\varepsilon =2\varepsilon \left({Cu}^{2+},{Cl}^{-}\right)+\varepsilon \left(Eph^{-},{Na}^{+}\right)-\varepsilon ({Cu}_{2}\left(Eph{)}^{3+},{Cl}^{-}\right)$$ (5)

$${Cu}_{2}\left(Eph\right){OH}^{2+};∆\varepsilon =2\varepsilon \left({Cu}^{2+},{Cl}^{-}\right)+\varepsilon {\left(Eph^{-},{Na}^{+}\right)}^{+}-\varepsilon \left({Cu}_{2}\left(Eph\right){OH}^{2+},{Cl}^{-}\right)–\varepsilon \left(H^{+},{Cl}^{-}\right)-0.015$$ (6)

$$Cu(Eph{)}_{2}{OH}^{-};∆\varepsilon =\varepsilon \left({Cu}^{2+},{Cl}^{-}\right)+2\varepsilon {\left(Eph^{-},{Na}^{+}\right)}^{+}-\varepsilon (Cu\left(Eph{)}_{2}{OH}^{-},{Na}^{+}\right)–\varepsilon \left(H^{+},{Cl}^{-}\right)-0.015$$ (7)

$${Cu}_{2}(Eph{)}_{2}^{2+};∆\varepsilon =2\varepsilon \left({Cu}^{2+},{Cl}^{-}\right)+2\varepsilon \left(Eph^{-},{Na}^{+}\right)-\varepsilon ({Cu}_{2}\left(Eph{)}_{2}^{2+},{Cl}^{-}\right)$$ (8)

where *k* (Cu(*Eph*)_2_^0^) is the Setschenow coefficient of the neutral species (Setschenow, 1889). If a ternary hydrolytic species is formed, the activity coefficient of water must be considered (log a_w_ = 0.015) in the calculation of the specific ion interaction parameter, as well as the specific ion interaction parameter ε(H^+^, Cl^−^).

## 3 Results

### 3.1 Epinephrine and alginate acid-base behavior

The acid-base properties of epinephrine has already been studied by the research group, which also studied the solubility and concentration of the ligand neutral species at different experimental conditions as well as the dependence on the ionic strength and temperature of thermodynamic parameters (Bretti et al., 2015) and commented on the literature attribution of equilibrium constants to epinephrine protonable groups (Antikainen and Witikainen, 1973).

The protonation behavior of the alginate polyelectrolyte was experimentally investigated by potentiometry at *I* = 0.15–1.00 mol dm^−3^ in NaCl_(aq)_ and *T* = 298.15K.

For the data elaboration of acid-base properties and metal complexation of polyelectrolytes, different approaches have been proposed and reported in the literature (Katchalsky, 1954; Högfeldt, 1988; Högfeldt et al., 1989) because the aqueous behavior of such high molecular weight ligands depends on factors such as ionic medium, ionic strength, electrostatic charge, temperature, and dissociation degree (α), thta as well known, depend on the pH of the solution.

Among the possible approaches, classical methods such as the modified Henderson-Hasselbalch equation proposed by Katchalsky (Katchalsky and Spitnik, 1947; Katchalsky, 1954) and the three parameters of the Högfeldt model (Högfeldt, 1988; Högfeldt et al., 1989) are particularly effective and accurate for describing the acid-base polyelectrolyte behavior in aqueous solutions as a function of α (Bretti et al., 2017). Unfortunately, employing the mentioned models require many calculations, and is often not simple for inexpert researchers.

In this light, a simplified approach, named the polyprotic-like model, was proposed, and reported in the literature (Crea et al., 2010). Briefly, this method allows a polyelectrolyte, in terms of acid-base and complexing ability, to be treated as a low molecular weight ligand, considering the minimum number of protonation sites useful to describe the system, independently of the dissociation degree. The polyprotic-like model was applied to many different classes of polyelectrolytes with various chemical structures, molecular weights, and functional groups. The data were compared with those obtained using the Henderson-Hasselbalch and Högfeldt models (Crea et al., 2010) and very similar results, without a significant loss of precision, were observed among the different approaches. A much simpler procedure for calculating the equilibrium constants characterized the data elaboration using the polyprotic-like model that was, therefore, considered a valid alternative to the classical equations for polyelectrolytes.

Based on all these considerations, the treatment of experimental data on alginate acid-base properties was carried out by applying the polyprotic-like model. In particular, the polyelectrolyte was assumed to behave as a low molecular weight ligand consisting of a monomeric unit of β-D-mannuronic acid and one of α-L-guluronic acid, with a total of two protonable sites, namely two carboxylic groups, and charge z = 2-. Alginate protonation constants, as reported in Table 2, were expressed by the following stepwise (Eq. 9) and overall (Eq. 10) equilibria:

$$H^{+}+H_{\left(r-1\right)}{\left(Alg\right)}^{-2+\left(r-1\right)}=H_{r}{\left(Alg\right)}^{-2+r}\;K_{r}^{H}$$ (9)

$${rH}^{+}+{Alg}^{2-}=H_{r}{\left(Alg\right)}^{\left(-2+r\right)}\beta_{r}^{H}$$ (10)

**TABLE 2:** Protonation constants of alginate (*Alg* ^2-^) at different ionic strengths in NaCl_(aq)_ and *T* = 298.15K.

*Ī*/mol dm^−3^	log*K* ^H^_1_ ^a^	log*β* ^H^_2_ ^b^	log*K* ^H^_2_ ^a^
0.144	3.150 ± 0.003^c^	5.486 ± 0.003	2.336
0.500	3.08 ± 0.01	5.35 ± 0.01	2.27
0.750	2.94 ± 0.01	5.08 ± 0.01	2.14
0.956	2.88 ± 0.01	5.124 ± 0.008	2.244

^a^ log*K* ^H^_r_, refers to equilibrium in Eq. 9.

^b^ log*β* ^H^_2_ refers to equilibrium in Eq. 10.

^c^ ±std. dev. Standard uncertainties: u(*T*) = 0.15K, u(*I*) = 0.001 mol dm^−3^.

As observable in Table 2, the protonation constants decrease with increasing ionic strength. Only in the case of log*K* ^H^_2_ can a slight reversal trend be observed at *I* = 0.956 mol dm^−3^, with respect to the other experimental conditions. The effect of this variable on the protonation of alginate can be also observed in Supplementary Figure S1. The H_2_(*Alg*)^0^_(aq)_ species reaches percentages of 66% and 57% at *I* = 0.144 and 0.956 mol dm^−3^, respectively, at pH ∼ 2.0. The formation of the H(*Alg*)^-^ is shifted towards more acidic pH values when the ionic strength increases, achieving percentages of 57% at pH ∼ 2.8 and 51% at pH ∼ 2.6, respectively. Starting from pH ∼ 5.0, the ligand is present as a free ligand, namely *Alg* ^2-^.

The results here obtained are comparable with those reported by De Stefano et al. (De Stefano et al., 2005). Authors used the same approach here employed for the determination of protonation constants, and at *I* = 0.50 mol dm^−3^ in NaNO_3(aq)_, they calculated the following values: log *K* ^H^_1_ = 3.135 and log*K* ^H^_2_ = 2.581. These data are in good agreement with the experimental alginate protonation constants in Table 2, determined at the same *I*/mol dm^−3^ and *T*/K conditions.

### 3.2 Hydrolysis of the metal cations and formation of uranyl/acetate complexes

The acid-base properties of Cu^2+^ and UO_2_^2+^ were already studied at *T* = 298.15K and *I* = 0.15–1.00 mol dm^−3^ in NaCl_(aq)_ (Baes and Mesmer, 1976; Gianguzza et al., 2004; Brown and Ekberg, 2016); these data are reported in Supplementary Tables S1, S2 of the Supplementary Information section.

Furthermore, since for the preparation of the dioxouranium(VI) (UO_2_^2+^) standard solutions the UO_2_(*Ac*)_2_ salt (*Ac*: acetate, *see* Materials and Method section) was also used and this metal cation tends to form stable complexes with acetate (Crea et al., 2003), the formation constants of the UO_2_^2+^/*Ac* ^−^ complexes were considered (see Supplementary Table S3 of the Supplementary Information section) as input in the speciation model of the UO_2_^2+^/*Eph* ^−^ and mixed Cu^2+^/UO_2_^2+^/*Eph* ^−^ mixed system.

### 3.3 Binary M^n+^/ligand systems

The elaboration of the experimental data on metal-ligand systems collected at *I* = 0.15–1.00 mol dm^−3^ in NaCl_(aq)_ and *T* = 298.15K using different analytical techniques led to the determination of metal/ligand complexes with different stoichiometry, owing to the various acid-base behaviors of ligands. The selection of the best speciation model was carried out applying some criteria already discussed in previous works (Irto et al., 2019b; Irto et al., 2020).

#### 3.3.1 Cu^2+^/Eph^−^ interaction

The study on the interaction of Cu^2+^ with epinephrine was carried out by potentiometric, UV-Vis spectrophotometric, calorimetric, and thermogravimetric techniques at *I* = 0.15–1.00 mol dm^−3^ in NaCl_(aq)_ and *T* = 298.15K.

Potentiometric data were collected in the pH range 3.0–8.5 up to the formation of sparingly soluble species. During the titrations, the measurement solutions assumed an intense dark red colour, which became clearer, to a light red-orange, with increasing the pH. The UV-Vis data, recorded at lower Cu^2+^ and *Eph* ^−^ concentrations than those used for potentiometry (*see* Materials and Methods section), were elaborated at pH 3.0–10.5 without the observation of precipitate in the measurement solutions. The best possible speciation scheme featured five species: Cu(*Eph*)_2_^0^_(aq)_, Cu(*Eph*)_2_OH^−^, Cu_2_(*Eph*)OH^2+^, Cu_2_(*Eph*)^3+^, and Cu_2_(*Eph*)_2_^2+^. The Cu(*Eph*)_2_OH^−^ complex was not determined at *I* = 0.732 mol dm^−3^ in NaCl_(aq)_.

The overall formation constants (Table 3) determined for these species are referred to the equilibria in Eqs 11, 12.

$${pCu}^{2+}+q{Eph}^{-}+{rH}^{+}={Cu}_{p}{\left(Eph\right)}_{q}H_{r}^{\left(2p+r-q\right)}\beta_{pqr}$$ (11)

$${pCu}^{2+}+qEph^{-}+{rH}_{2}O={Cu}_{p}{\left(Eph\right)}_{q}{\left(OH\right)}_{r}^{\left(2p-r-q\right)}+{rH}^{+}\beta_{pq-r}$$ (12)

**TABLE 3:** Experimental and suggested overall formation constants of Cu^2+^/*Eph* ^−^ species at different ionic strengths in NaCl_(aq)_ and *T* = 298.15K.

*I*/mol dm^−3^	log*β*_Cu(*Eph*)2_ ^a^	log*β*_Cu(*Eph*)2OH_ ^b^	log*β*_Cu2(*Eph*)OH_ ^b^	log*β*_Cu2(*Eph*)_ ^a^	log*β*_Cu2(*Eph*)2_ ^a^
0.156	19.75^d^±0.04^c^	11.18 ± 0.03	8.77 ± 0.03	13.88 ± 0.02	25.53 ± 0.002
0.156	19.86^e^±0.04	11.35 ± 0.03	8.99 ± 0.03	13.95 ± 0.02	25.527 ± 0.002
0.156	19.76^f^±0.05	11.25 ± 0.05	8.93 ± 0.05	13.90 ± 0.03	25.51 ± 0.05
0.494	18.92 ± 0.02	10.69 ± 0.03	9.89 ± 0.01	14.72 ± 0.01	25.02 ± 0.06
0.494	19.19 ± 0.02	10.73 ± 0.02	9.90 ± 0.01	14.83 ± 0.01	25.445 ± 0.006
0.494	19.15 ± 0.03	10.69 ± 0.03	9.87 ± 0.02	14.81 ± 0.02	25.44 ± 0.03
0.732	18.53 ± 0.02	-	10.60 ± 0.01	15.46 ± 0.02	25.56 ± 0.01
0.732	18.84 ± 0.02	-	10.60 ± 0.01	15.45 ± 0.02	25.49 ± 0.01
0.732	18.84 ± 0.03	-	10.60 ± 0.01	15.45 ± 0.01	25.49 ± 0.02
0.971	19.05 ± 0.03	10.09 ± 0.02	11.36 ± 0.02	16.10 ± 0.01	25.58 ± 0.01
0.971	18.50 ± 0.03	10.03 ± 0.02	11.32 ± 0.02	16.07 ± 0.01	25.57 ± 0.01
0.971	18.55 ± 0.05	10.05 ± 0.02	11.35 ± 0.02	16.09 ± 0.02	25.57 ± 0.03

^a^ log*β*_pqr_ refers to equilibrium in Eq. 11.

^b^ log*β*_pq-r_ refers to equilibrium in Eq. 12.

^c^ ±std. dev.

^d^ from potentiometry.

^e^ from spectrophotometry.

^f^ suggested values calculated at all ionic strength conditions by means of the minimization of the error square sum of potentiometric and UV-Vis spectrophotometric data. Standard uncertainties: u(*T*) = 0.15 K, u(*I*) = 0.001 mol dm^−3^.

As observable in Table 3, the obtained data are in good agreement between the two analytical techniques. This allowed for the calculation of the Cu^2+^/*Eph* ^−^ species and by using the minimization of the error square sum (De Stefano et al., 1997) on the data obtained from the two techniques, it was possibile to calculate the corresponding “suggested” values.

It is evident that the Cu^2+^/*Eph* ^*-*^ complexes have high stability, allowing their formation also at low concentrations (i.e., from spectrophotometric data).

The possible formation of 1:2 complexes was already reported in different investigation on the Cu^2+^/*Eph* ^−^ system (Jameson and Neillie, 1965; Jameson and Neillie, 1966; Grgas-Kužnar et al., 1974; Rahaman and Korenkiewicz, 1976; Gergely et al., 1981; Kiss and Gergely, 1983; Materazzi et al., 2002; Al-Ayed et al., 2013; Krasnovskaya et al., 2020); the high complexes stability of the species can be interpreted taking into account the coordination that occurs through the phenolic groups of epinephrine rather than involving the side chain (Jameson and Neillie, 1965; Jameson and Neillie, 1966).

**FIGURE 2:**
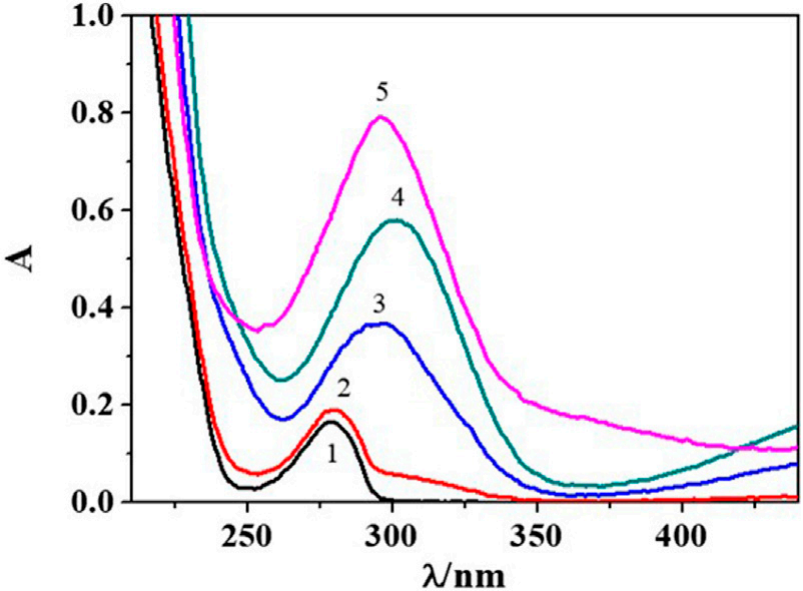
UV-Vis spectra recorded at some pH values for the Cu^2+^/*Eph* ^−^ system at *I* = 0.156 mol dm^−3^ in NaCl_(aq)_, *T* = 298.15K, *c*_Cu2+_ = 0.0566 mmol dm^−3^ e *c*_Eph-_ = 0.0622 mmol dm^−3^. 1. pH = 3.07; 2. pH = 5.26; 3. pH = 6.48; 4. pH = 7.49; 5. pH = 10.73.

From the analysis of UV-Vis spectra in Figure 2 at *I* = 0.156 mol dm^−3^, it is possible to observe an absorption band at λ_max_ = 280 nm and pH ∼ 3.1 which significantly increases in intensity with the rise in pH. Furthermore, a bathochromic shift occurs in the pH range ∼ 6.5–7.5 with λ_max_ = 303 nm, providing possible evidence of the formation of metal-ligand species. Then, at pH > 10.5, a hypsochromic shift can be observed with λ_max_ = 296 nm, possibly owing to epinephrine oxidation/degradation processes occurring at this experimental condition. For this reason, absorbance values recorded at more alkaline pH were not considered during data treatment.

The distribution of Cu^2+^/*Eph* ^−^ species can be investigated by means of the distribution diagrams in Supplementary Figure S2, drawn at *I* = 0.156 and 0.971 mol dm^−3^ using potentiometric data. Significant differences between the two ionic strengths can be observed in terms of percentages and pH of formation, in particular for the Cu_2_(*Eph*)^3+^, Cu_2_(*Eph*)OH^2+^, and Cu_2_(*Eph*)_2_^2+^ species. At *I* = 0.156 mol dm^−3^, the main complex determined in the pH range of many natural waters and biological fluids (pH ∼ 5.0–8.2) are the Cu(*Eph*)_2_^0^_(aq)_ and Cu_2_(*Eph*)_2_^2+^, while at *I* = 0.971 mol dm^−3^, the predominant species are the Cu_2_(*Eph*)OH^2+^ and the Cu(*Eph*)_2_^0^_(aq)_.

The effect of temperature on Cu^2+^/*Eph* ^*-*^ speciation was investigated by performing isoperibolic calorimetric titrations at *T* = 298.15K and *I* = 0.50 mol dm^−3^. The enthalpy change values for the protonation of *Eph* ^*-*^ (Bretti et al., 2015), hydrolysis of Cu^2+^ (Brown and Ekberg, 2016), and the dissociation of water (De Stefano et al., 2001) were taken from the literature.

**FIGURE 3:**
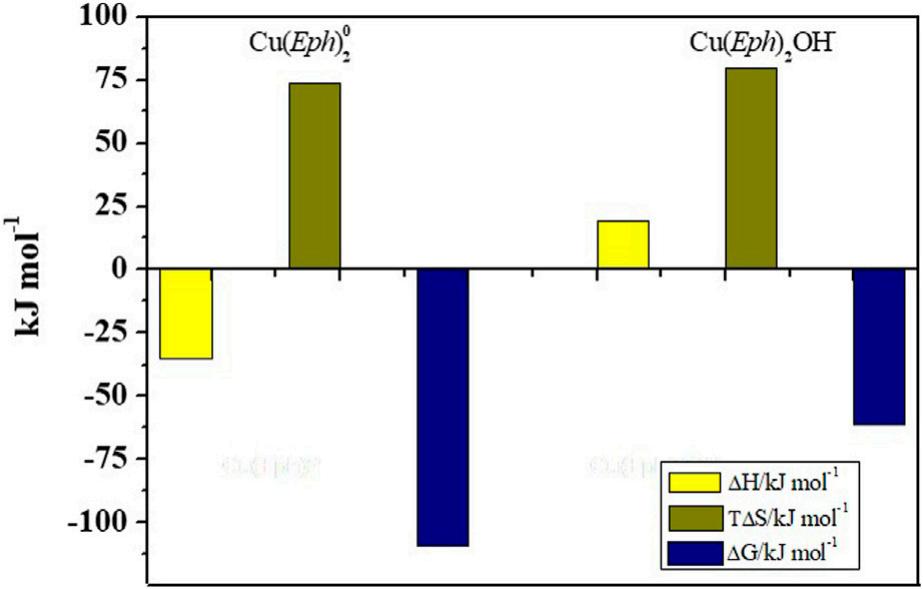
Bar plot for the enthalpy and entropy change values of the Cu^2+^/*Eph* ^−^ system species at *I* = 0.50 mol dm^−3^ and *T* = 298.15 K.

The determination of the enthalpy change values of the Cu^2+^/*Eph* ^−^ species formation was limited by the low ligand solubility at the mentioned ionic strength condition (log*S* = ∼ −2.07) (Bretti et al., 2015), which did not allow the preparation of more concentrated solutions. For this reason, it was possible to experimentally calculate the enthalpy of formation of only two complexes, namely, Cu(*Eph*)_2_^0^_(aq)_ and Cu(*Eph*)_2_OH^−^. The formation of the Cu(*Eph*)_2_^0^_(aq)_ species was favored by an exothermic contribution (Δ*H*_Cu(*Eph*)2_ = −35 ± 1 kJ mol^−1^), whilst the Cu(*Eph*)_2_OH^−^ was favoured by an endothermic (Δ*H*_Cu(*Eph*)2OH_ = 19 ± 4 kJ mol^−1^) contribution (see also Figure 3).

The reliability of the obtained results is also supported by the low values of both the standard deviation for the global fit of experimental data (σ = 0.018) and the mean deviation of the variation of the heats of reaction ( ∂ Q = 0.151).

The entropic contribution (*T*Δ*S*_Cu(*Eph*)2_ = 74 ± 3 kJ mol^−1^, *T*Δ*S*_Cu(*Eph*)2OH_ = 80 ± 10 kJ mol^−1^) was the driving force for the Cu^2+^/*Eph* ^−^ complexes formation, and both the processes were spontaneous, as highlighted by the negative values of the calculated free Gibbs energy (Δ*G*_Cu(*Eph*)2_ = −109.16 ± 0.16, Δ*G*_Cu(*Eph*)2OH_ = −60.83 ± 0.05).

**FIGURE 4:**
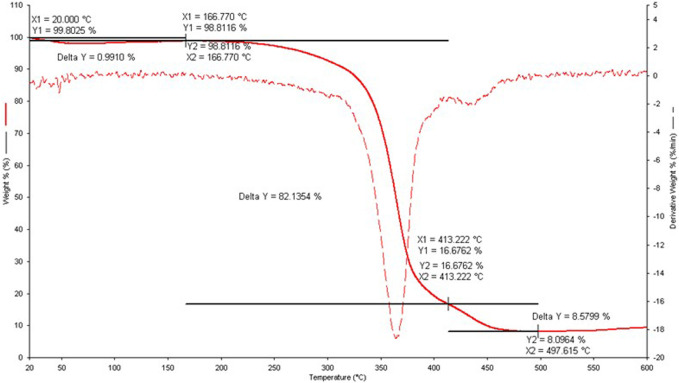
Thermogravimetric curve for the Cu^2+^/*Eph* ^−^ solid sample obtained at *I* = 0.156 mol dm^−3^, *c*_Cu2+_ = 1.0 mmol dm^−3^ and *c*_*Eph-*_ = 4.0 mmol dm^−3^. Solid line: % weight loss as function of *t/°*C, dash line: derivative weight loss vs. *t/°*C.

In addition, the precipitates collected at the end of the potentiometric titrations were characterized by thermogravimetry (TGA) to determine the precipitate stoichiometry and gain information on the thermal stability. Three decomposition processes were observed, as reported in Figure 4.

The first decomposition process was characterized by a weight loss of 0.99%, significantly lower with respect to the two subsequent ones of 82.13% and 8.58%, respectively. For each process, the corresponding molecular weights of fragments lost were 9.81 g mol^−1^, 813.90 g mol^−1^, and 84.90 g mol^−1^, respectively. The residual of the decomposition process at 79.50 g mol^−1^ was assumed to be CuO (MW = 79.55 g mol^−1^). At the investigated conditions, the most probable stoichiometry for the precipitate formation was the 1:5 one, namely the Cu(*Eph*)_5_ species. This result is highly dependent on the component concentrations and molar ratios employed to prepare the samples.

#### 3.3.2 UO_2_^2+^/Eph^−^ system

The binding ability of epinephrine towards dioxouranium(VI) had been already experimentally investigated by the research group using the same experimental conditions (i.e., temperature, ionic medium, ionic strength, and component concentration) as selected for the present study (Crea et al., 2020). The speciation model featured the following metal/ligand species: UO_2_(*Eph*)^+^, UO_2_(*Eph*)OH^0^_(aq)_; (UO_2_)_2_(*Eph*)_2_^2+^ and (UO_2_)_2_(*Eph*)_2_(OH)_2_^0^_(aq)_. The dependence on *I*/mol dm^−3^ was modelled by means of a Debye-Hückel type equation and Specific ion Interaction Theory (SIT). By means of isoperibolic calorimetric titrations, the enthalpy change values of species formation were determined.

#### 3.3.3 Cu^2+^/Alg^2-^ system

The elaboration of potentiometric data was performed in the pH range 3.0–10.0 due to the formation of sparingly soluble species occurring at pH ∼ 2.5–2.8. Owing to the low solubility of alginate in acid solutions, before the titration, the pH was corrected up to ∼10.5 and then the resulting solutions titrated with standard solution of HCl. The formation of precipitate was observed at pH values dependent on the experimental conditions (component concentration and ionic strength). Applying the above mentioned selection criteria, the best speciation model was obtained considering the following species: Cu(*Alg*)^0^_(aq)_, Cu(*Alg*)OH^−^, Cu(*Alg*)(OH)_2_^2-^, Cu(*Alg*)(OH)_3_^3-^, and Cu(*Alg*)_2_^2-^. The overall equilibrium constants determined for these species referred to the equilibria in Eqs. 13, 14:

$${Cu}^{2+}+q{\left(Alg\right)}^{2-}=Cu{\left(Alg\right)}_{q}^{\left(2-2q\right)}\beta_{1q}$$ (13)

$${Cu}^{2+}+{Alg}^{2-}+{rH}_{2}O=Cu\left(Alg\right){\left(OH\right)}_{r}^{\left(-r\right)}+{rH}^{+}\beta_{\mathrm{11}-r}$$ (14)

In Table 4, the equilibrium constants obtained at *I* = 0.15–1.00 mol dm^−3^ in NaCl_(aq)_ and *T* = 298.15K are reported; we can observe an increase of the overall stability constants (log *β*) with an increase in the ionic strength.

**TABLE 4:** Experimental overall formation constant of Cu^2+^/*Alg* ^2-^ species at different ionic strengths in NaCl_(aq)_ and *T* = 298.15K.

*Ī*/mol dm^−3^	log*β*_Cu(*Alg*)_ ^a^	log*β*_Cu(*Alg*)OH_ ^b^	log*β*_Cu(*Alg*)(OH)2_ ^b^	log*β*_Cu(*Alg*)(OH)3_ ^b^	log*β*_Cu(*Alg*)2_ ^a^
0.149	3.09 ± 0.08^c^	-3.32 ± 0.04	-9.79 ± 0.03	-20.03 ± 0.04	5.53 ± 0.10
0.500	3.23 ± 0.10	-3.10 ± 0.06	-9.49 ± 0.06	-19.36 ± 0.05	6.10 ± 0.12
0.750	3.28 ± 0.09	-2.99 ± 0.06	-9.39 ± 0.06	-19.24 ± 0.05	6.36 ± 0.10
0.984	3.35 ± 0.15	-3.00 ± 0.10	-9.35 ± 0.10	-19.48 ± 0.11	6.47 ± 0.06

^a^ log*β* refers to equilibrium in Eq. 13.

^b^ log*β* refers to equilibrium in Eq. 14.

^c^ ±std. dev. Standard uncertainties: u(*T*) = 0.15 K, u(*I*) = 0.001 mol dm^−3^.

De Stefano et al. (De Stefano et al., 2010) reported the results of a potentiometric study carried out using alginic acid and ISE-[Cu^2+^] and ISE-[H^+^] electrodes, at *I* = 0.098–0.727 mol dm^−3^ in NaNO_3_ ionic medium and *T* = 298.15K, but at different metal (*c*_Cu_^2+^ = 0.25–0.35 mmol dm^−3^) and ligand (*c*_*Alg*_^2-=^4.51–4.62 mmol dm^−3^) concentrations, as well as pH range (4.1–4.7). The authors determined only one complex species, namely, the Cu(*Alg*)^0^_(aq)_, whose stability constants at *I* = 0.10 mol dm^−3^ were: log*β*_110_ = 3.626 and 3.586, respectively. Some differences can be observed among these data and the value (log*β*_110_ = 3.09 ± 0.08) presented in Table 4 at *I* = 0.149 mol dm^−3^, possibly due to the different speciation models and experimental conditions.

**FIGURE 5:**
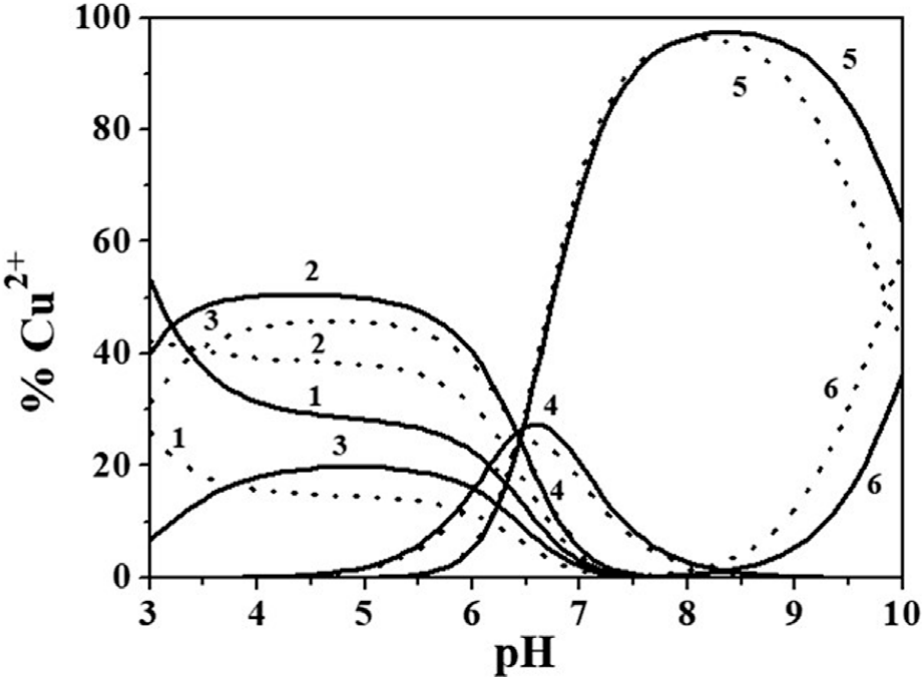
Distribution diagram of Cu^2+^/*Alg* ^2-^ species at *I* = 0.149 (solid line) and 0.984 (dotted line) mol dm^−3^, *c*_cu2+_ = 0.70 mmol dm^−3^, *c*_*Alg2-*_ = 2.10 mmol dm^−3^. Species: 1. Free Cu^2+^; 2. Cu(*Alg*)^0^_(aq)_; 3. Cu(*Alg*)_2_^2-^; 4. Cu(*Alg*)OH^−^; 5. Cu(*Alg*)(OH)22^−^; 6. Cu(*Alg*)(OH)_3_^3-^.

The distribution of Cu^2+^/*Alg* ^2-^ species can be investigated by the analysis of distribution diagrams in Figure 5, drawn at *I* = 0.149 and 0.984 mol dm^−3^.

All the species reach formation percentages between 21% and 97%. The main complexes at physiological pH (pH ∼ 7.4) and at the pH of seawater (pH ∼ 8.1) are the Cu(*Alg*)OH^−^ and Cu(*Alg*)(OH)_2_^2-^.

#### 3.3.4 Dependence on ionic strength of thermodynamic parameters

The dependence on ionic strength of alginate protonation constants, Cu^2+^/*Eph* ^−^ and Cu^2+^/*Alg* ^2-^ complexes formation constants was modelled using an extended Debye-Hückel type equation and Specific ion Interaction Theory (SIT) approach (Biederman, 1975; Biederman, 1986). More details on these models are reported in Section 2.4. Models for ionic strength dependence. For both the binary systems, the stability constants at infinite dilution and the C empirical parameters are reported in Supplementary Tables S4–S6 together with the calculated values at *I* = 0.15–1.00 mol dm^−3^ in NaCl_(aq)_, allowing for their prediction in experimental conditions of real systems like biological fluids or natural waters.

Applying the same extended Debye-Hückel type equation (Eq. 1a) and Section 2.4.), De Stefano et al. (De Stefano et al., 2010) determined for the Cu(*Alg*)^0^_(aq)_ species the formation constants at infinite dilution (log^T^ *β*) and the parameter for the dependence on *I*/mol dm^−3^; the values they obtained are: log^T^ *β* = 5.05 and *C* = 0.69.

In the case of the Cu^2+^/*Eph* ^−^ system, the dependence of the formation constants on ionic strength was also investigated by means of the Specific ion Interaction Theory (SIT) approach, which requires the conversion of the concentrations and stability constants from the molar (mol dm^−3^) to the molal (mol kg^−1^_H2O_) concentration scale and the knowledge of the specific ion interaction parameters (ε) for all the ion-pairs involved in the complex formation equilibria. The values of 0.12 (Bretti et al., 2006), -0.219 (Bretti et al., 2015), and 0.08 (Grenthe and Puigdomenech, 1997) were used for the ε (H^+^,Cl^−^), ε (*Eph* ^−^,Na^+^), and ε (Cu^2+^,Cl^−^) parameters, respectively. For the equilibria that involve H_2_O molecules in the formation of the Cu^2+^/*Eph* ^−^ complexes, the activity coefficient of water (a_w_ = -0.015⋅*I*; valid in NaCl_(aq)_ at *T* = 298.15K) was also used (Foti et al., 2002).

The results of the calculation of the specific ion interaction parameter of the ionic species and of the Setschenow coefficient of the neutral one (*k* (Cu(*Eph*)_2_^0^)) (Setschenow, 1889) are reported in Table 5.

**TABLE 5:** Specific ion interaction parameters for the Cu^2+^/*Eph* ^−^ species at *T* = 298.15K.

Species	Parameters	Determined values
Cu(*Eph*)_2_^0^_(aq)_	*k* (Cu(*Eph*)_2_^0^)	0.34 ± 0.02^a^
Cu_2_(*Eph*)^3+^	ε (Cu_2_(*Eph*)^3+^,Cl^−^)	-2.703 ± 0.004
Cu_2_(*Eph*)OH^2+^	ε (Cu_2_(*Eph*)OH^2+^,Cl^−^)	-3.66 ± 0.01
Cu(*Eph*)_2_OH^−^	ε (Cu(*Eph*)_2_OH^−^,Na^+^)	0.41 ± 0.02
Cu_2_(*Eph*)_2_^2+^	ε (Cu_2_(*Eph*)_2_^2+^,Cl^−^)	-0.95 ± 0.01

^a^ ±std. dev.

### 3.4 Binary Eph^−^/Alg^2-^ system

Since *Eph* ^−^ behaves like a zwitterion and its secondary amine group is protonated up to pH∼10, we decided to investigate the possible ligand-ligand interaction with alginate, by means of potentiometric titration carried out at *I* = 0.146 mol dm^−3^ in NaCl_(aq)_ and *T* = 298.15K. Tests performed at the ionic range *I* = 0.50–1.00 mol dm^−3^ displayed the formation of precipitate in the measurement solution at the starting pH of ∼10.0, not allowing us to carry out experiments at the mentioned experimental conditions. A comparison between the titration curves of the *Eph* ^−^, *Alg* ^2-^ and *Eph* ^−^/*Alg* ^2-^ systems at *I* = 0.146 mol dm^−3^ is reported in Supplementary Figure S3, where some differences can be observed due to the different ligands’ acid-base behaviors and the formation of *Eph* ^−^/*Alg* ^2-^ binary species along the investigated pH range.

The experimental data collected in the pH range 2.0–10.0 were processed as already done for the other systems, and the best results were obtained for (*Eph*)(*Alg*)H^2–^, (*Eph*)(*Alg*)^3-^, (*Eph*)_2_(*Alg*)H_2_^2-^, and (*Eph*)(*Alg*)_2_^5-^. The overall stability constants were determined considering the general equilibrium in Eq. 15:

$$p{Eph}^{-}+q{Alg}^{2-}+{rH}^{+}={\left(Eph\right)}_{p}{\left(Alg\right)}_{q}H_{r}^{\left(-p+r-2q\right)}\beta_{pqr}$$ (15)

The stability constants (±std. dev.) at the indicated experimental conditions were: log*β*_(*Eph*)(*Alg*)H_ = 13.63 ± 0.05, log*β*_(*Eph*)(*Alg*)_ = 3.65 ± 0.05, log*β*_(*Eph*)2(*Alg*)H2_ = 27.95 ± 0.02, and log*β*_(*Eph*)(*Alg*)2_ = 6.43 ± 0.08.

**FIGURE 6:**
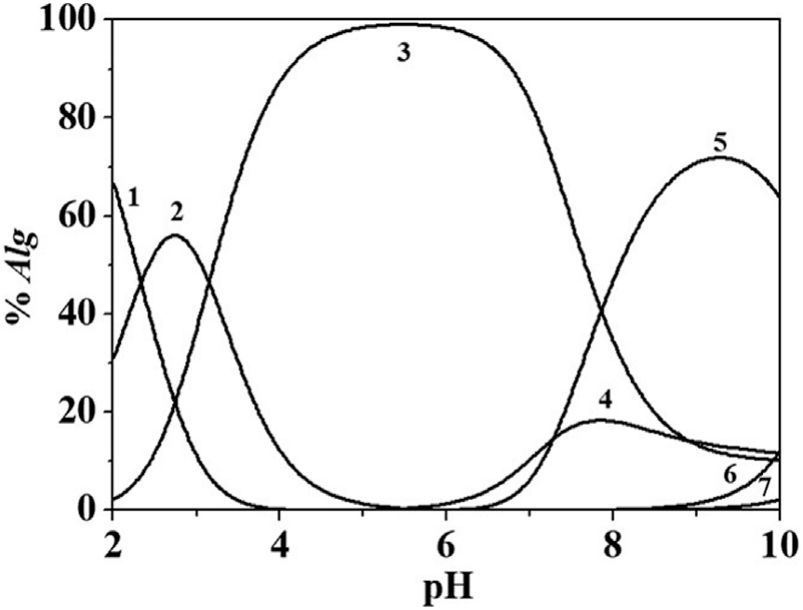
Distribution diagram of *Eph-/Alg* ^2-^ species at *I* = 0.146 mol dm^−3^ in NaCl_(aq)_, *T* = 298.15K, *c*_*Eph-*_ = 3.00 mmol dm^−3^; *c*_*Alg*2-_ = 1.50 mmol dm^−3^. Species: 1. H_2_(Alg)^0^_(aq)_; 2. H(*Alg*)^-^; 3. (*Alg*)^2-^; 4. (*Eph*)(*Alg*)H^2-^; 5. (*Eph*)_2_(*Alg*)H_2_^2-^; 6. (*Eph*)(*Alg*)^3-^; 7. (*Eph*)(*Alg*)^2(5-)^.

Figure 6 reports a distribution diagram of the *Eph* ^−^/*Alg* ^2-^ species at *I* = 0.146 mol dm^−3^ and *T* = 298.15K.

From the distribution diagram in Figure 6, it is possible to observe that the interaction between the two ligands start at pH ∼ 5.5. Up to pH ∼ 5.0, alginate is partially protonated and over this pH the formation of the *Eph* ^*-*^/*Alg* ^*2-*^ species occurs. At the experimental condition of Figure 6, the main species is the (*Eph*)_2_(*Alg*)H_2_^2-^ that reaches about 80% of formation at pH ∼ 9. The yield of formation of the other species reaches values of ∼10–20%.

To gain further information about this ligand-ligand system, UV-Vis spectrophotometric and Diffusion-Ordered NMR SpectroscopY (DOSY NMR) was carried out.

The UV-Vis spectra recorded for epinephrine showed a similar profile than the one reported in the literature at the same temperature, *c*_*Eph*_^*-*^ = 0.11 mmol dm^−3^ and *I* = 0.15 mol dm^−3^ in NaCl_(aq)_ (Bretti et al., 2015). In the case of alginate, a weak absorbance (A ≤ 0.05) was observed at the selected experimental conditions.

**FIGURE 7:**
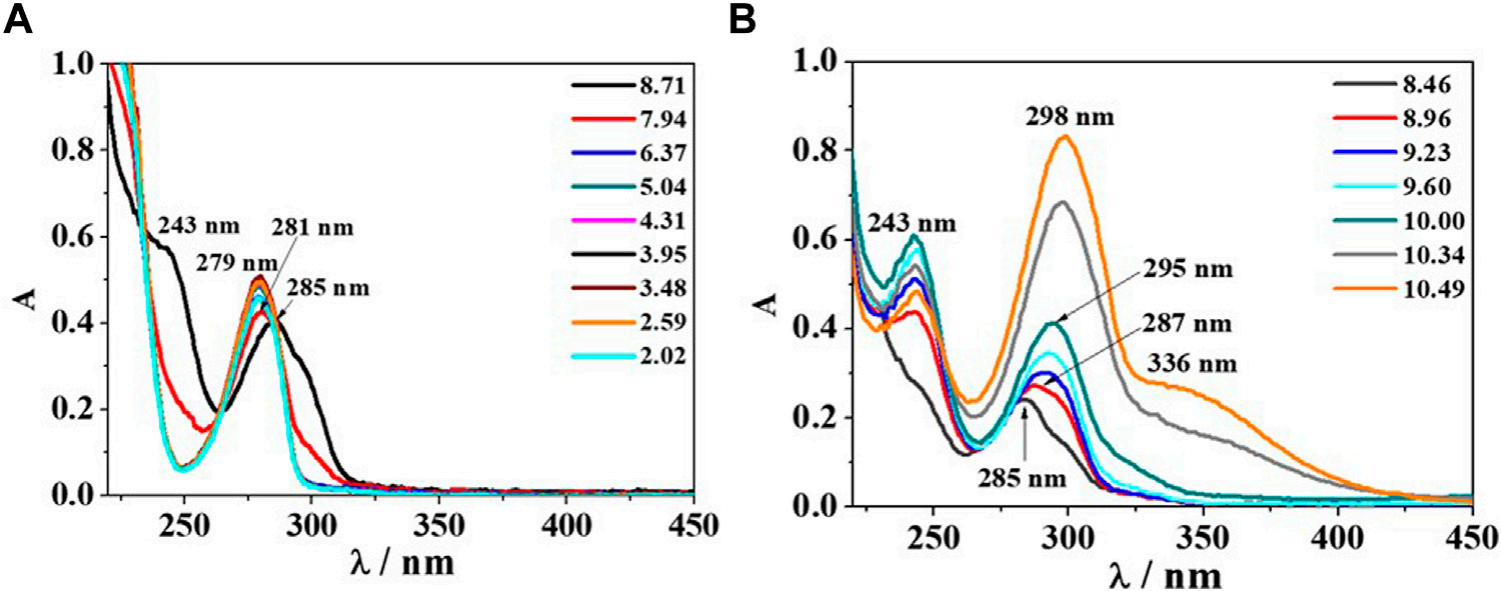
UV-Vis spectra of *Eph* ^−^/*Alg* ^2-^ system at *c*_*Eph-*_ = *c*_*Alg* ^2-^_ = 0.12 mmol dm^−3^, *T* = 298.15K and different pH values; **(A)** UV-Vis scans recorded in the pH range 8.71–2.02; **(B)** UV-Vis scans recorded in the pH range 8.46–10.49.

The analysis of UV-Vis spectra of the binary *Eph* ^−^/*Alg* ^2-^ system has been divided in two diagrams; the first one for the pH range 8.70–2.00 (Figure 7A), where an absorption band with λ_max_ = 285 nm and a shoulder at λ = 243 nm at pH ∼ 8.70 was observed. The band features a slight hypsochromic shift at λ_max_ = 281 nm and pH ∼ 7.9 and another one at λ_max_ = 279 nm in the pH range ∼ 5.0–2.6 with absorbance increase. Then, at pH ∼ 2.0, the signal intensity decreases, and no other shifts are observed. The second graph, at alkaline conditions (pH range 8.50–10.50, Figure 7B), highlights a bathochromic shift and an absorbance increase. The mentioned band with λ_max_ = 285 nm moves at λ_max_ = 287, 295, and 298 nm at pH ∼ 9.0, 10.0, and 10.5, respectively, while the shoulder, already observed in Figure 7A, becomes a resolved band with λ_max_ = 243 nm, increasing in intensity with pH. Furthermore, at pH ∼ 10.5, another shoulder is formed at λ = 336 nm.

The elaboration of the UV-Vis data collected in absence of ionic medium allowed us to obtain the same speciation model determined at *I* = 0.146 mol dm^−3^. The formation constant values (±std. dev.) obtained were: log*β*_(*Eph*)(*Alg*)H_ = 13.54 ± 0.25, log*β*_(*Eph*)(*Alg*)_ = 3.834 ± 0.003, log*β*_(*Eph*)2(*Alg*)H2_ = 28.35 ± 0.10, and log*β*_(*Eph*)(*Alg*)2_ = 6.892 ± 0.002. Considering the different experimental conditions in term of ionic strength and ligands concentration with respect to the ones selected to perform the potentiometric measurements, the mentioned data can be considered in good accordance with the ones determined at *I* = 0.146 mol dm^−3^.

To gain additional information about the interactions between *Eph* ^*–*^ and *Alg* ^*2–*^, the mixture was subjected to a series of NMR experiments. Simple mixing of the two compounds in D_2_O (*T* = 298.15K, pH ∼ 9.5) did not result in significant complexation-induced shifts of the resonances of epinephrine, providing no evidence of direct interaction. Analysis of the alginate resonances proved to be less straightforward, as they appear in ^1^HNMR spectra as broad overlapped multiplets (Belattmania et al., 2020).

**FIGURE 8:**
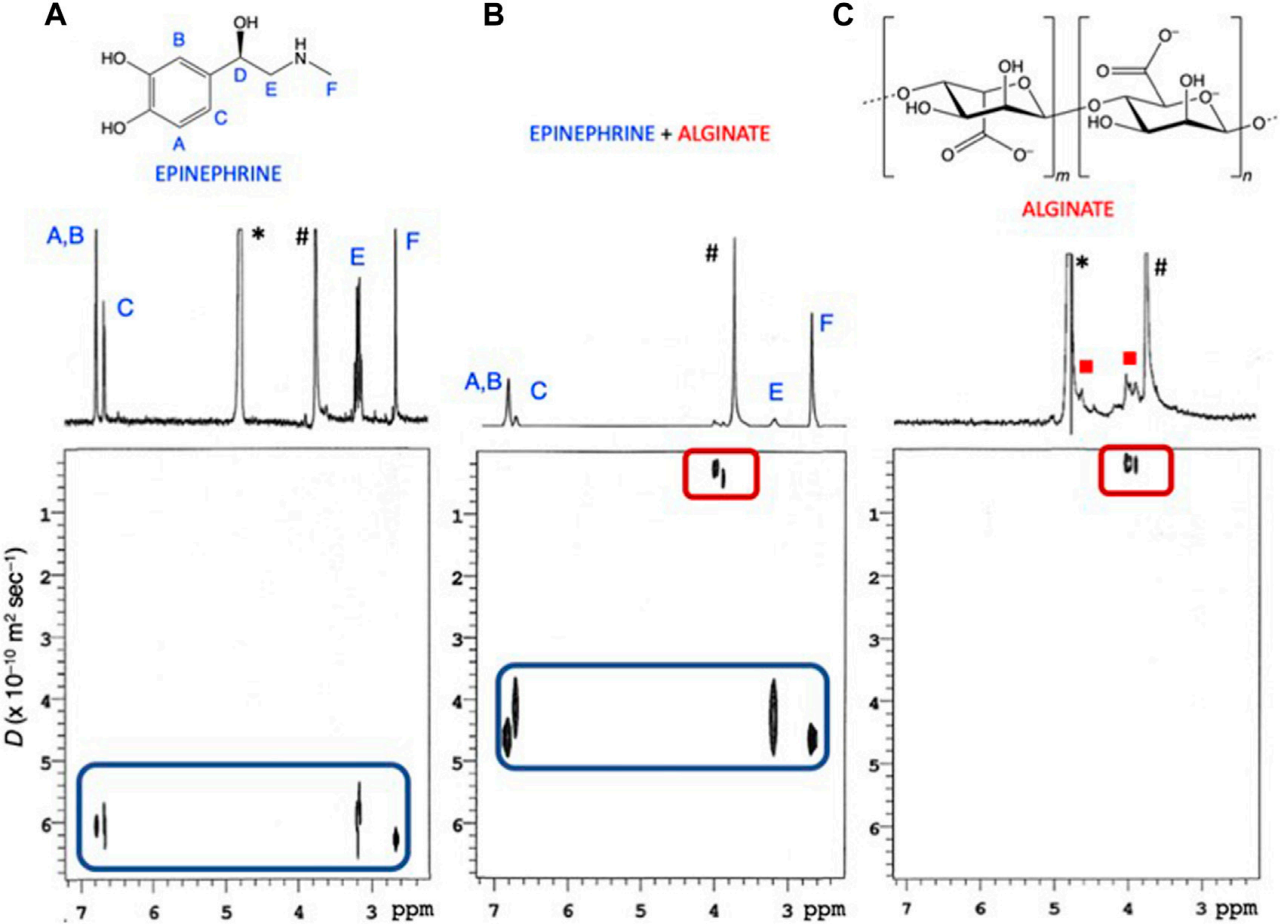
DOSY plots (500 MHz, *T* = 298.15 K, D_2_O) recorded on solutions containing: **(A)** *c*_*Eph-*_ = 2.29 mmol dm^−3^; **(B)** *c*_*Eph-*_ = 2.29 mmol dm^−3^ and *c*_*Alg*2*-*_ = 2.60 mmol dm^−3^; **(C)** *c*_*Alg*2*-*_ = 2.52 mmol dm^−3^ * Residual HOD solvent peak; ^#^1,4-dioxane internal standard.

Further insight was therefore gained by Diffusion-Ordered NMR SpectroscopY (DOSY NMR) experiments on separate solutions of epinephrine and sodium alginate, as well as on a mixture of the two components. DOSY experiments provide self-diffusion coefficients (*D*) of dissolved species that may be used also for the detection of intermolecular interactions (Gattuso et al., 2014; Barbera et al., 2015). Spectra recorded on separate solutions of *Eph* ^*–*^ and *Alg* ^*2–*^ (Figures 8A–C) allowed for the extraction of the self-diffusion coefficient of the free species, *D*_*Eph*_ *-*_(free)_ and *D*_*Alg^2-^*_ _(free)_, which were found to be 6.05 ± 0.216 and 0.224 ± 0.0945 (× 10^−10^ m^2^ s^−1^), respectively, in perfect agreement with the very different molecular size of the two species (i.e., unimolecular *Eph* ^*–*^ *vs.* polymeric *Alg* ^*2–*^). Upon admixing the two compounds (Figure 8B), DOSY analysis showed that alginate maintained its unaltered self-diffusion coefficient, whereas epinephrine significantly decreased to 4.41 ± 0.207 and 0.262 ± 0.111 (× 10^−10^ m^2^ s^−1^) for *D*_*Eph*_^*-*^_(obs)_ and *D*_*Alg*_^2-^_(obs)_, respectively, indicating that, in the *Eph* ^*–*^/*Alg* ^*2–*^ aggregates, the translational mobility of faster-diffusing *Eph* ^*–*^ is reduced as a consequence of its binding to slower-diffusing *Alg* ^*2–*^ polymer.

The diffusion coefficients obtained by DOSY experiments, in fast exchanging (on the NMR time-scale) systems, are weighted averages of all the free and bound species present at given pH/concentration values. As a result, *D* values for free and bound species can be used to estimate the mole fraction (χ) of *Eph* ^*–*^ bound to the larger *Alg* ^*2–*^, according to Eq. 16:

$$D_{Eph}{-}_{\left(obs\right)}=\chi D_{Eph}{-}_{\left(bound\right)}+\left(1–\chi \right)D_{Eph}{-}_{\left(free\right)}$$ (16)

where *D*_*Eph*_ *-*_(obs)_ is the weighted average of the diffusion coefficients measured in the *Eph* ^*–*^ + *Alg* ^*2–*^ sample and *D*_*Eph*_ *-*_(free)_ is the coefficient measured for *Eph* ^*–*^ alone. As for *D*_*Eph*_ *-*_(bound)_, when considering the association of a small molecule, such as *Eph* ^*–*^, to a much larger one (e.g., a polymer), the *D* value for the fully bound *Eph* ^*–*^ can be considered to be very close to that of *Alg* ^*2–*^ alone. As a consequence, the equation *D*_*Eph*_ *-*_(bound)_ = *D*_*Alg2*_ *-*_(free)_ can be safely assumed, ultimately yielding a bound fraction value χ = 0.28.

Taking into account the concentration of *Eph* ^*–*^ and *Alg* ^*2–*^ in the DOSY experiment (*c*_*Eph*_^−^ = 2.29 and *c*_*Alg*_^2-^ = 2.60 mmol dm^−3^, respectively), a simple calculation allowed us to deduce a log*K* value of 2.298 for *Eph* ^*–*^/*Alg* ^*2–*^ association.

This data can be considered in good agreement with those obtained from potentiometric and UV-spectrophotometric measurements, taking into account that the log*K* value obtained from the DOSY experiments is valid at that condition.

### 3.5 Ternary systems

#### 3.5.1 Cu^2+^/Eph^−^/Alg^2-^ interactions

The investigation on the ternary metal-ligand-ligand system were performed at *I* = 0.146 mol dm^−3^ in NaCl_(aq)_, *T* = 298.15K, different components concentrations, and metal:ligands molar ratios. To avoid the formation of sparingly soluble species, the solutions were prepared with an excess of epinephrine, since from measurements carried out with an excess of alginate, the precipitate formed at pH ∼ 10, excluding the possibility of carrying out the experiments.

The data processing was performed in the pH range 3.0–10.0 because the formation of precipitate at pH ∼ 2.7 did not allow us to continue the experiments.

In Supplementary Figure S4, it is possible to appreciate an example of the color variation of the analysis solution during one of the measurements. At pH ∼ 10.0 the solution containing the metal and the two ligands assumed a yellow colour, becoming orange tending to red at pH ∼ 7.2, and then bright red at ∼ pH 6.5–6.8.

The elaboration of experimental data led to the determination of a speciation model featured by the following ternary species: Cu(*Eph*)(*Alg*)^-^, Cu(*Eph*)(*Alg*)H^0^_(aq)_, Cu(*Eph*)_2_(*Alg*)^2-^, Cu(*Eph*)_2_(*Alg*)H^−^, and Cu(*Eph*)(*Alg*)_2_H^2-^ whose formation can be expressed by Eq. 17:

$${Cu}^{2+}+p{Eph}^{-}+qAlg^{2-}+{rH}^{+}=Cu{\left(Eph\right)}_{p}{\left(Alg\right)}_{q}H_{r}^{\left(2-p-2q+r\right)}\beta_{1pqr}$$ (17)

The formation constant values (±std. dev.) determined for the mentioned ternary species were: log*β*_Cu(*Eph*)(*Alg*)_ = 15.81 ± 0.01, log*β*_Cu(*Eph*)(*Alg*)H_ = 21.35 ± 0.02, log*β*_Cu(*Eph*)2(*Alg*)_ = 24.55 ± 0.01, log*β*_Cu(*Eph*)2(*Alg*)H_ = 30.75 ± 0.04, and log*β*_Cu(*Eph*)(*Alg*)2H_ = 24.84 ± 0.02.

The analysis of the distribution diagram in Supplementary Figure S5 shows that, in slight epinephrine excess, the ternary species with the highest formation percentages are the Cu(*Eph*)(*Alg*)^-^, Cu(*Eph*)_2_(*Alg*)^2-^, and Cu(*Eph*)_2_(*Alg*)H^−^, reaching 33%, 47%, and 46% at pH ∼ 7.9, 9.0, and 4.3, respectively. At the mentioned conditions, the formation of binary Cu^2+^/ligands complexes is mainly observed in the pH range 5.0–10.0. The most significant binary Cu^2+^/*Eph* ^−^ species are the Cu_2_(*Eph*)_2_^2+^ and Cu(*Eph*)_2_OH^−^ which reaching 30% and 37% at pH ∼ 6.2 and 10.0, respectively. Concerning the Cu^2+^/*Alg* ^*2-*^ complexes, the hydrolytic Cu(*Alg*)(OH)_2_^2-^ and Cu(*Alg*)(OH)_3_^3-^ species forms at pH > 7.5 and exceeds 25% and 10% at pH ∼ 9.3 and 10.0, respectively. On the basis of these results, it is evident that a correct speciation study in multicomponent solutions cannot exclude the possible formation of mixed ternary complexes.

Based on these observations, in Supplementary Figure S6, a comparison between the sum of the mmoles of the metal/ligands species, considering and ignoring the ternary complexes, is shown.

The main differences between the two curves are found in the pH range 3.0–8.0, where the total contribution of the formed mixed species is much higher with respect to those given by the binary ones. On the contrary, at pH > 8.0, the only formed ternary species is the Cu(*Eph*)_2_(*Alg*)^2-^ and the Cu^2+^/*Eph* ^−^ and Cu^2+^/*Alg* ^2-^ complexes prevail.

In the literature, Beck and Nagypal (Beck and Nagypál, 1991) asserted that the formation of hetero-metallic or ternary metal-ligand-ligand species is possible and statistically favored with respect to the corresponding homo-metallic or binary M^n+^/*L*_1_^z−^ and M^n+^/*L*_2_^v−^ (M^n+^ = metal cation, *L*_1_^z−^, *L*_2_^v−^ = ligands with different charge) complexes with the same stoichiometry. They suggested the possibility of the calculation of the “extra-stability” for the mixed ternary species using two approaches, namely, by means of the calculation of experimental (log*X*_exp_) and statistical (log*X*_stat_) extra-stability constants (Beck and Nagypál, 1991) and stated that if log*X*_exp_ > log*X*_stat_, the formation of mixed complexes is thermodynamically favored.

As an application of this principle, a further analysis of the speciation schemes of Cu^2+^/*Eph* ^−^ (Table 3) and Cu^2+^/*Alg* ^2-^ (Table 4) systems suggested the presence of a common Cu*L*_2_ species, namely, the Cu(*Eph*)_2_^0^_(aq)_ and Cu(*Alg*)_2_^2-^, comparable with the Cu(*Eph*)(*Alg*)^-^ complex displaying the same stoichiometry. Considering the indications reported in the literature, the log*X*_exp_ can be expressed considering the following equilibrium (Eq. 18):

$$Cu{\left(Eph\right)}_{2_{\left(aq\right)}}^{0}+Cu{\left(Alg\right)}_{2}^{2-}=2Cu\left(Eph\right){\left(Alg\right)}^{-}X_{\exp}$$ (18)

By performing a linear combination of the formation constants of binary and ternary complexes, it was possible to obtain:

$$\log X_{\exp}=2\log\beta_{Cu\left(Eph\right)\left(Alg\right)}–\;\log\beta_{Cu\left(Eph\right)2}–\;\log\beta_{Cu\left(Alg\right)2}=6.34$$

Once the experimental extra-stability was determined, the corresponding statistical value (*X*_stat_) was computed using the expression:

$$X_{stat}={\left[h!/\left(i!\cdot j!\right)\right]}^{h}$$ (19)

where h, i, and j are the stoichiometric coefficients in Eq. 18, namely, 1, 1, and 2, respectively. The *X*_stat_ and log*X*_stat_ were calculated and resulted to be 4.00 and 0.60, respectively.

The comparison among the determined experimental and statistical extra-stability constant values suggested that log*X*_exp_ > log*X*_stat_, confirming that the formation of ternary complexes is not only possible, but thermodynamically favored over the binary ones. The formation of the mixed ternary complexes and the extra-stability are very important factors both from an environmental and biological point of view, since in many cases, the extra-stability of the mixed ternary complexes favored the mobility and transport of these complexes, owing to an increase in the metal’s solubility.

#### 3.5.2 *Cu* ^*2+*^ */*UO_2_^2+^ */Eph* ^−^ *system*

The elaboration of potentiometric data recorded at *I* = 0.160 mol dm^−3^ in NaCl_(aq)_, *T* = 298.15K, led to the determination of a speciation model featured by three ternary species, such as: Cu(UO_2_)(*Eph*)^3+^, Cu(UO_2_)(*Eph*)_2_^2+^, and Cu(UO_2_)(*Eph*)_2_OH^+^. The overall formation constants, determined considering the equilibria in Eqs. 20, 21, were: log*β*_Cu(UO2)(*Eph*)_ = 15.44 ± 0.01, log*β*_Cu(UO2)(*Eph*)2_ = 27.82 ± 0.01, and log*β*_Cu(UO2)(*Eph*)2OH_ = 22.61 ± 0.02:

$${Cu}^{2+}+{{UO}_{2}}^{2+}+q{Eph}^{-}=Cu\left({UO}_{2}\right){\left(Eph\right)}_{q}^{\left(4-q\right)}\beta_{11q}$$ (20)

$${Cu}^{2+}+{{UO}_{2}}^{2+}+2{Eph}^{-}+H_{2}O=Cu\left({UO}_{2}\right){\left(Eph\right)}_{2}{OH}^{+}+H^{+}\;\beta_{112-1}$$ (21)

The calculation of log*X*_exp_ and log*X*_stat_ values was performed to verify also whether the hetero-metallic Cu^2+^/UO_2_^2+^/*Eph* ^−^ species formation could be thermodynamically favored with respect to the homo-metallic Cu^2+^/*Eph* ^−^ (Table 3) and UO_2_^2+^/*Eph* ^−^ (Crea et al., 2020) complexes. The stability of the species with analogous stoichiometry to be analysed were the binary Cu_2_(*Eph*)_2_^2+^ and (UO_2_)_2_(*Eph*)_2_^2+^ with respect to the mixed Cu(UO_2_)(*Eph*)_2_^2+^. The calculation of log*X*_exp_ value referred to the following equilibrium (Eq. 22):

$${Cu}_{2}{\left(Eph\right)}_{2}^{2+}+{\left({UO}_{2}\right)}_{2}{\left(Eph\right)}_{2}^{2+}=2Cu\left({UO}_{2}\right){\left(Eph\right)}_{2}^{2+}X_{\exp}$$ (22)

The experimental extra-stability constant was calculated as follows:

$$\log X_{\exp}=2\log\beta_{Cu\left(UO2\right)\left(Eph\right)2}–\;\log\beta_{\left(UO2\right)2\left(Eph\right)2}–\;\log\beta_{Cu2\left(Eph\right)2}=2.79$$

while the statistical value was determined using Eq. 19, with *X*_stat_ = 4.00 and log*X*_stat_ = 0.60.

The calculated log*X*_stat_ was lower with respect to the log*X*_exp_, confirming the Beck and Nagypal (Beck and Nagypál, 1991) assertation that the formation of hetero-metallic complexes is thermodynamically favored over the homo-metallic ones.

As further evidence of the mixed species significance, in Supplementary Figure S7, a comparison between the sum of the metals-ligand complexes formation percentages, considering and ignoring the ternary species, is shown. Up to pH ∼ 4.0–4.5, the two curves do not deviate from each other, while as the pH increases, significant changes in the sum of the formation percentages are observed, owing to the formation of the three ternary complexes with a ΔΣ of ∼30%.

### 3.6 Sequestering ability

The evaluation of the sequestering ability of a ligand towards one or more metal cations assumes a particular importance for the resolution of real biological and environmental issues, such as the treatment of human body for detoxification or the remediation of polluted sites, respectively, both involving the use of a chelating agent.

For this purpose, the calculation of an empirical parameter, the pL_0.5_, representing the total ligand concentration required for the sequestration of the 50% of a metal cation present at trace (c_M_^n+^ ∼ 10^–12^ mol dm^−3^) concentration in solution, was proposed by our research group (Crea et al., 2014). This objective and quantitative parameter can be described by a sigmoidal-type Boltzmann equation, with asymptotes equal to one for pL→-∞ and 0 for pL → +∞ (Eq. 23):

$$x_{M}=\frac{1}{1+10^{\left(pL-pL_{0.5}\right)}}$$ (23)

with:

*x*_M_ = mole fraction of M^n+^ complexed by the ligand; pL = -log*c*_L_; pL_0.5_ = -log*c*_L_, if *x*_M_ = 0.5.

The higher the pL_0.5_ value, the stronger the sequestering ability of a ligand towards the selected metal cation.

The evaluation of the sequestering ability of epinephrine and alginate towards Cu^2+^ was carried out by means of the calculation of the pL_0.5_ at *T* = 298.15K, different ionic strengths in NaCl_(aq)_, and pH values using the equilibrium constants determined from potentiometric data (Tables 3, 4). From the analysis of the values in Table 6 and the graphs in Supplementary Figure S8, it can be concluded that for both the ligands, the sequestering ability increases with pH raising, possibly owing to the gradual epinephrine and alginate deprotonation, favouring the Cu^2+^/ligands electrostatic interaction.

**TABLE 6:** pL_0.5_ values calculated using Eq. 23 at *T* = 298.15K, different ionic strengths in NaCl_(aq)_, and pH conditions.

*Ligand*	*I/*mol dm^−3^	pH	pL_0.5_	*Ligand*	*I/*mol dm^−3^	pH	pL_0.5_
*Eph* ^-^	0.15	6.5	3.88	*Eph* ^-^	0.50	7.2	5.13
	0.15	7.4	5.26		0.75	7.2	5.02
	0.15	8.1	7.11		1.00	7.2	5.22
*Alg* ^2-^	0.15	3.0	2.76	*Alg* ^2-^	0.75	7.4	5.40
	0.15	4.0	3.12		1.00	3.0	3.24
	0.15	5.0	3.19		1.00	4.0	3.50
	0.15	6.0	3.32		1.00	5.0	3.54
	0.15	7.4	5.00		1.00	6.0	3.67
	0.15	8.1	6.18		1.00	7.4	5.43
	0.15	9.0	7.25		1.00	8.1	6.62
	0.15	10.0	7.71		1.00	9.0	7.72
	0.50	7.4	5.30		1.00	10.0	8.22

Concerning the effect of ionic strength, for both *Eph* ^−^ and *Alg* ^2-^, the pL_0.5_ values calculated at pH ∼ 7.4, respectively, similar to the physiological value, follow the same trend of the stability constants reported in Tables 3, 4.

The pL_0.5_ calculation was a very efficient tool for the investigation of the sequestering ability of many ligands towards metal cations. Nevertheless, if, in a multicomponent system, one or more polynuclear complexes were determined, like in the case of the Cu^2+^/*Eph* ^*-*^ investigation where Cu_2_(*Eph*)OH^2+^, Cu_2_(*Eph*)^3+^, and Cu_2_(*Eph*)_2_^2+^ species were obtained, they should not form at the trace (c_M_^n+^ ∼ 10^–12^ mol dm^−3^) concentration in which the empirical parameter is usually computed. In these cases, the comparison of the sequestering ability of a ligand towards two or more metals or two ligands towards a metal cation can fail, since the pL_0.5_ value calculated for the metal:ligand systems that have in the speciation model polynuclear species, cannot be effectively representative of the quantification of the binding ability. In this light, objective comparisons among the affinity of the ligands towards Cu^2+^ can be performed by determining the pM parameter (Raymond and Carrano, 1979) (see 3.7. Cu^2+^ affinity section), considering the free metal cation concentration for the calculations, without being affected by the presence of complexes with various stoichiometry.

### 3.7 Cu^2+^ affinity

The abovementioned drawbacks, observable in the calculation of the pL_0.5_ parameter for making a comparison between epinephrine and alginate affinity towards Cu^2+^ owing to the presence of polynuclear complexes in one of the systems, may be solved by means of the pM (Raymond and Carrano, 1979) parameter, in this case, the pCu, with pCu = -log [Cu]_free_ and *c*_Cu_^2+^ = 0.001 mmol dm^−3^. The calculations were performed at *c*_*Eph*_^−^ = *c*_*Alg*_^2-^ = 0.01 mmol dm^−3^ and at different pHs, namely, pH ∼ 7.4 to simulate physiological conditions and pH ∼ 8.1 for seawater.

**FIGURE 9:**
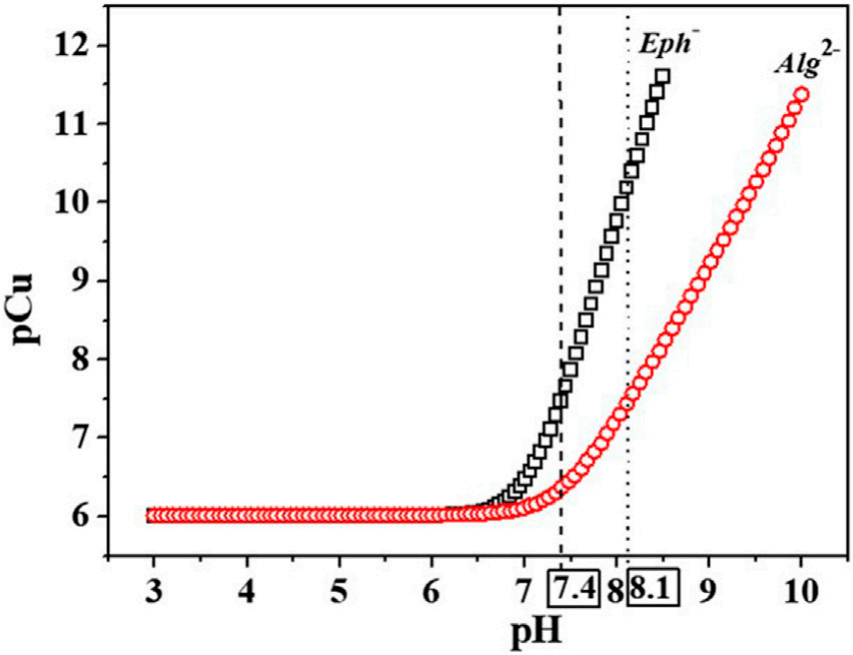
pCu values trend calculated vs. pH for the Cu^2+^/*Eph-* and Cu^2+^/*Alg* ^2-^ systems. pCu = -log [Cu]_free_ with *c*_cu2+_ = 0.001 mmol dm^−3^ and *c*_*Eph-*_ = *c*_*Alg2-*_ = 0.01 mmol dm^−3^.

The pCu values were calculated for the two Cu^2+^/*Eph* ^−^ and Cu^2+^/*Alg* ^2-^ systems at *I* = 0.15 and 1.00 mol dm^−3^ in NaCl_(aq)_, *T* = 298.15K, and compared to each other to evaluate which of the ligands could have the best efficiency towards the metal cation. As observable in Supplementary Table S7 and Figure 9, at all experimental conditions, epinephrine displays a higher Cu^2+^ affinity than alginate. This tendency could be possibly explainable looking at the “*hard*-*soft* acids and bases” theory (HSAB) (Pearson, 1963; Pearson, 1968), where interactions between *hard* acids and *hard* bases as well as *soft* acids and *soft* bases are kinetically and thermodynamically favored when compared with *hard*-*soft* interactions. As a consequence of these considerations, *borderline* acids and *borderline* bases interactions should be favored too. On this basis, the affinity between Cu^2+^, a *borderline* metal cation, and the *borderline* adrenaline amino group could be higher with respect to the *hard* functional groups (-COOH) present in the alginate structure. Both the ligands also featured other *hard* sites, namely, -OH groups.

The analysis of data in Supplementary Table S7 also suggests that the metal efficacy increases with pH, possibly due to the decrease of free copper(II) in solution in favor of the complexed form with the ligands binding groups, and with ionic strength increase, as already observed from the calculation of the pL_0.5_ values.

As a comparison, Supplementary Figure S9 reports the correlation between the pL_0.5_ values and pH for the Cu^2+^/*Alg* ^*2-*^ system at two different ionic strengths, *I* = 0.15 and 1.00 mol dm^−3^, respectively, where it is possible to observe a very similar trend to the one obtained from the pCu calculation.

## 4 Conclusion and discussions

The speciation of epinephrine (*Eph* ^−^) in the presence of alginate (*Alg* ^2-^) and two biological and environmental relevant metal cations (Cu^2+^, UO_2_^2+^) was investigated at *T* = 298.15K, *I* = 0.15–1.00 mol dm^−3^ in NaCl_(aq)_. The formation of binary and ternary complexes was evaluated and, since epinephrine can behave as a zwitterion in aqueous solution, the possible *Eph* ^−^/*Alg* ^2-^ interaction was also studied.

The main results can be summarized as follows:

a) The equilibrium constants for alginate protonation and for the complexation with Cu^2+^ were determined by potentiometry and modelled for the dependence on ionic strengths using an extended Debye-Hückel type equation;
b) The complexing ability of epinephrine towards the metal cation was studied by means of potentiometry and UV-Vis spectrophotometry; the formation constants were in agreement among the two analytical techniques and the effect of ionic strength on the stability of the species was studied using an extended Debye-Hückel type equation and the Specific ion Interaction Theory (SIT) approach;
c) Isoperibolic titration calorimetry allowed us to determine the enthalpy change values of formation for only two Cu^2+^/*Eph* ^−^ complexes, owing to the solubility problems of epinephrine at the selected experimental conditions; the entropic contribution was the driving force for the Cu^2+^/*Eph* ^−^ species formation;
d) Thermogravimetric experiments gained information on the thermal stability and metal/ligand complexes stoichiometry;
e) The sequestering ability of *Eph* ^−^ and *Alg* ^2-^ towards Cu^2+^, evaluated by the pL_0.5_ calculation, increases with pH and ionic strength, and the calculation of the pM parameter confirmed that *Eph* ^−^ has a higher metal affinity with respect to *Alg* ^2-^ at selected pH and *I*/mol dm^−3^ conditions;
f) The formation of binary *Eph* ^−^/*Alg* ^2-^ species was tested using UV-Vis spectrophotometry and ^1^H NMR measurements; the obtained results are in very good agreement with those determined from potentiometry both in term of speciation model and stability of the ligand-ligand species; moreover, the DOSY measurements allowed us to calculate a mean stability constant for the interaction between *Eph* ^−^ and *Alg* ^2-^, log*K*_*Eph*_^−^_/*Alg*_^2-^ = 2.9. Taking into account that this stability constant can be considered as mean value of all the interactions between the component at those experimental condition, this data can also be considered reliable to quantify the interaction between the two ligands;
g) The ternary Cu^2+^/*Eph* ^−^/*Alg* ^*2-*^ and Cu^2+^/UO_2_^2+^/*Eph* ^−^ interactions were investigated by potentiometry and different speciation schemes were determined; the knowledge of the possible formation of ternary species is fundamental for the treatment of many real biological and environmental problems;
h) The extra-stability constants of selected Cu^2+^/*Eph* ^−^/*Alg* ^*2-*^ and Cu^2+^/UO_2_^2+^/*Eph* ^−^ species were calculated and their formation were more thermodynamically favorable than the corresponding binary ones with the same stoichiometry.

## Data Availability

The original contributions presented in the study are included in the article/Supplementary Material, further inquiries can be directed to the corresponding author.
