# Supplementary material for: Binary and ternary complexes of epinephrine with alginate and biologically and environmentally relevant metal cations
Source: Front Chem. 2023 Apr 25;11:1189308. doi: 10.3389/fchem.2023.1189308 (PMC10167000; doi:10.3389/fchem.2023.1189308)
Supplement: Supplementary file 1 [file DataSheet1.docx]

**Supplementary information**

**Binary and ternary complexes of epinephrine with alginate and biologically and environmentally relevant metal cations**

**Tables**

Table S1. Hydrolytic constants of Cu^2+^ (Baes and Mesmer, 1976; Brown and Ekberg, 2016) at different ionic strengths in NaCl_(aq)_ and *T* = 298.15 K.

| *I*/mol dm^-3^ | log*β*_Cu(OH)_^a)^ | log*β*_Cu(OH)2_^a)^ | log*β*_Cu(OH)3_^a)^ | log*β*_Cu(OH)4_^a)^ | log*β*_Cu2(OH)2_^a)^ |
| --- | --- | --- | --- | --- | --- |
| 0.15 | -8.23 | -17.48 | -27.80 | -39.09 | -10.61 |
| 0.50 | -8.29 | -17.59 | -27.82 | -38.87 | -10.72 |
| 0.75 | -8.30 | -17.62 | -27.83 | -38.79 | -10.76 |
| 1.00 | -8.29 | -17.65 | -27.83 | -38.73 | -10.80 |

^a)^ log*β*_pr_ related to the equilibrium: pCu^2+^ rH_2_O = Cu_p_(OH)_r_^(2p-r)^ + rH^+^.

Table S2. Hydrolytic constants of UO_2_^2+^ (Gianguzza et al., 2004) at different ionic strengths in NaCl_(aq)_ and *T* = 298.15 K.

| *I*/mol dm^-3^ | log*β*_(UO2)OH_^a)^ | log*β*_(UO2)2(OH)2_^a)^ | log*β*_(UO2)3(OH)4_^a)^ | log*β*_(UO2)3(OH)5_^a)^ | log*β*_(UO2)3(OH)7_^a)^ |
| --- | --- | --- | --- | --- | --- |
| 0.15 | -5.50 | -6.02 | -12.27 | -16.64 | -29.72 |
| 0.50 | -5.72 | -6.14 | -12.40 | -16.95 | -29.84 |
| 0.75 | -5.84 | -6.19 | -12.41 | -17.05 | -29.86 |
| 1.00 | -5.96 | -6.24 | -12.40 | -17.13 | -29.85 |

^a)^ log*β*_pr_ related to the equilibrium: pUO_2_^2+^ + rH_2_O = (UO_2_)_p_(OH)_r_^(2p-r)^ + rH^+^.

Table S3. Formation constants of UO_2_^2+^/*Ac*^-^ species (Crea et al., 2003) at different ionic strengths in NaCl_(aq)_ and *T* = 298.15 K.

| *I*/mol dm^-3^ | log*β*_(UO2)_*_Ac_*^a)^ | log*β*_(UO2)_*_Ac_*_2_^a)^ | log*β*_(UO2)_*_Ac_*_3_^a)^ | log*β*_(UO2)_*_Ac_*_3(OH)_^b)^ |  |
| --- | --- | --- | --- | --- | --- |
| 0.15 | 2.44 | 4.02 | 6.58 | 1.92 |  |
| 0.50 | 2.38 | 4.09 | 6.44 | 1.79 |  |
| 0.75 | 2.36 | 4.02 | 6.43 | 1.77 |  |
| 1.00 | 2.33 | 4.24 | 6.40 | 1.78 |  |

^a)^log*β*_pq_ refer to the equilibrium: pUO_2_^2+^ + q*Ac*^-^ = (UO_2_)_p_*Ac*_q_^(2p-q)^; ^b)^log*β*_pq-r_ refer to the equilibrium: pUO_2_^2+^ + q*Ac*^-^ + rH_2_O = (UO_2_)_p_*Ac*_q_(OH)_r_^(2p-q-r)^ + r H^+^.

Table S4**.** Thermodynamic parameters for the dependence on ionic strength of alginate protonation at infinite dilution in NaCl_(aq)_ and *T* = 298.15K.

| Species | *z** | log^T^*β*^a)^ | *C*^b)^ | *I*/mol dm^-3^ | | | | |
| --- | --- | --- | --- | --- | --- | --- | --- | --- |
|  |  |  |  | 0.15 | 0.25 | 0.50 | 0.75 | 1.00 |
| H(*Alg)*^-^ | 4 | 3.634±0.006^c)^ | 0.09±0.01 | 3.148 | 3.075 | 2.981 | 2.936 | 2.912 |
| H_2_(*Alg)*^0^_(aq)_ | 6 | 6.226±0.006 | 0.006±0.008 | 5.477 | 5.353 | 5.178 | 5.077 | 5.007 |

^a)^ log^T^*β*^H^_r_ referred to equilibrium in eq. (10); ^b)^ empirical parameter calculated by means of eq. (1a); ^c)^ ±std. dev. Standard uncertainties: u(*t*) = 0.15K, u(*I*) = 0.001 mol dm^-3^.

Table S5. Thermodynamic parameters for the dependence on ionic strength of Cu^2+^/*Eph*^-^ species at infinite dilution and calculated formation constants at different ionic strengths and *T* = 298.15K.

| Species | *z** | log^T^*β* | *C*^c)^ | *I*/mol dm^-3^ | | | | |
| --- | --- | --- | --- | --- | --- | --- | --- | --- |
|  |  |  |  | 0.15 | 0.25 | 0.50 | 0.75 | 1.00 |
| Cu(*Eph*)_2_^0^_(aq)_^a)^ | 6 | 20.66±0.07^d)^ | -0.92±0.11^d)^ | 19.77 | 19.56 | 19.15 | 18.81 | 18.51 |
| Cu(*Eph*)_2_OH^- b)^ | 4 | 11.93±0.06 | -1.10±0.08 | 11.26 | 11.07 | 10.68 | 10.34 | 10.01 |
| Cu_2_(*Eph*)OH^2+ b)^ | 4 | 8.92±0.06 | 3.34±0.07 | 8.92 | 9.17 | 9.89 | 10.66 | 11.44 |
| Cu_2_(*Eph*)^3+ a)^ | 0 | 13.49±0.04 | 2.68±0.05 | 13.88 | 14.15 | 14.82 | 15.49 | 16.16 |
| Cu_2_(*Eph*)_2_^2+ a)^ | 6 | 26.17±0.06 | 0.64±0.08 | 25.51 | 25.45 | 25.44 | 25.50 | 25.59 |

^a)^ log^T^*β*_pqr_ related to equilibrium in eq. (11); ^b)^ log*β*_pq-r_ referred to equilibrium in eq. (12); ^c)^ empirical parameter calculated by means of eq. (1a); ^d)^ ±std. dev. Standard uncertainties: u(*t*) = 0.15K, u(*I*) = 0.001 mol dm^-3^.

Table S6. Thermodynamic parameters for the dependence on ionic strength of formation constants of Cu^2+^/*Alg*^2-^ species in NaCl_(aq)_ and *T* = 298.15K.

| Species | *z** | log^T^*β* | *C*^c)^ | *I*/mol dm^-3^ | | | | |
| --- | --- | --- | --- | --- | --- | --- | --- | --- |
|  |  |  |  | 0.15 | 0.25 | 0.50 | 0.75 | 1.00 |
| Cu(*Alg*)^0^_(aq)_^a)^ | 8 | 4.25±0.11^d)^ | 0.76±0.30^d)^ | 3.512 | 3.447 | 3.464 | 3.578 | 3.734 |
| Cu(*Alg*)OH^- b)^ | 6 | -2.48±0.06 | 0.86±0.14 | -2.999 | -3.027 | -2.960 | -2.820 | -2.649 |
| Cu(*Alg*)(OH)_2_^2- b)^ | 2 | -9.42±0.05 | 0.56±0.10 | -9.485 | -9.463 | -9.362 | -9.237 | -9.102 |
| Cu(*Alg*)(OH)_3_^3- b)^ | -4 | -20.16±0.04 | 0.19±0.10 | -19.529 | -19.418 | -19.230 | -19.091 | -18.973 |
| Cu(*Alg*)_2_^2- a)^ | 8 | 6.71±0.10 | 1.57±0.14 | 6.033 | 6.023 | 6.179 | 6.433 | 6.728 |

^a)^ log^T^*β*_pqr_ related to equilibrium in eq. (13); ^b)^ log*β*_pq-r_ referred to equilibrium in eq. (14); ^c)^ empirical parameter calculated by means of eq. (1a); ^d)^ ±std. dev. Standard uncertainties: u(*t*) = 0.15K, u(*I*) = 0.001 mol dm^-3^.

Table S7. pCu values calculated for the Cu^2+^/*Eph*^-^ and Cu^2+^/*Alg*^2-^ systems.

| *Ligand* | *I/*mol dm^-3^ | pH | pCu | *Ligand* | *I/*mol dm^-3^ | pH | pCu |
| --- | --- | --- | --- | --- | --- | --- | --- |
| *Eph*^-^ | 0.15 | 7.4 | 7.46 | *Alg*^2-^ | 0.15 | 7.4 | 6.36 |
|  |  | 8.1 | 10.18 |  |  | 8.1 | 7.43 |
|  | 1.00 | 7.4 | 8.08 |  | 1.00 | 7.4 | 6.59 |
|  |  | 8.1 | 9.72 |  |  | 8.1 | 7.81 |

**Figures**


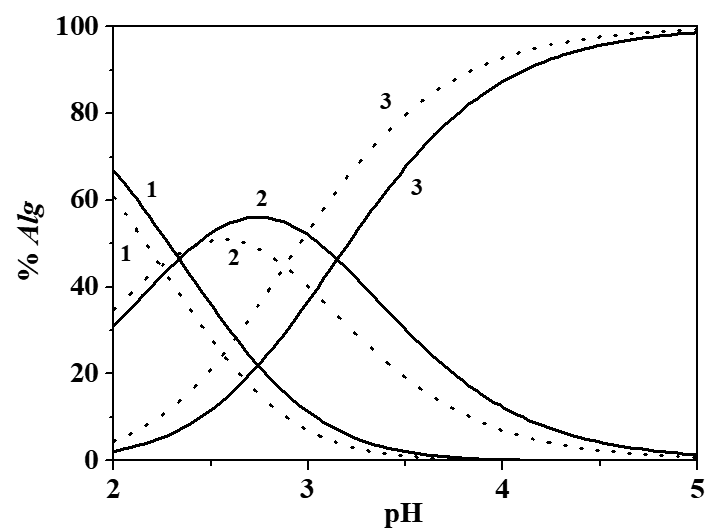


Figure S1. Distribution diagrams of alginate species at *I* = 0.144 (solid line) and 0.956 (dotted line) mol dm^-3^ in NaCl_(aq)_, *T* = 298.15K, *c_Alg_*_2-_ = 1.0 mmol dm^-3^. Species: 1. H_2_(*Alg*)^0^_(aq)_; 2. H(*Alg*)^-^; 3. (*Alg*)^2-^.

**
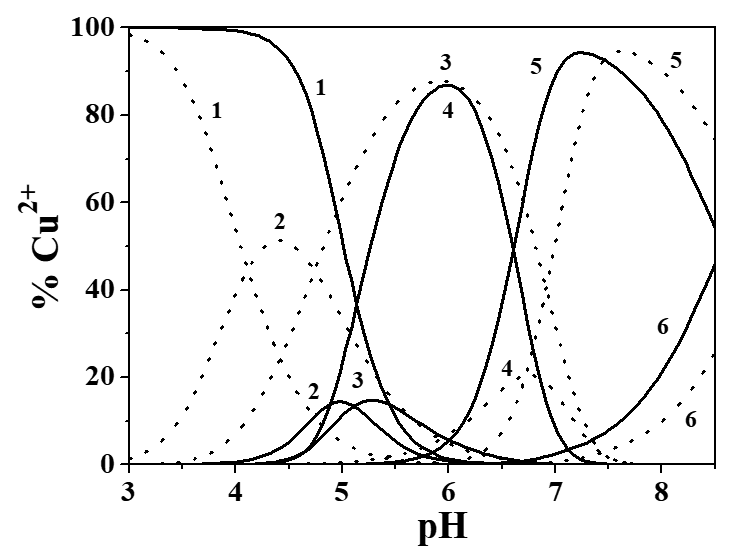
**

Figure S2. Distribution diagrams of Cu^2+^/*Eph*^-^ species at *I* = 0.156 (solid line) and 0.971 (dotted line) mol dm^-3^ in NaCl_(aq)_, *T* = 298.15K, *c*_Cu2+_ = 1.0 mmol dm^-3^, *c_Eph_*_-_ = 3.0 mmol dm^-3^. Species: 1. free Cu^2+^; 2. Cu_2_(*Eph*)^3+^; 3. Cu_2_(*Eph*)OH^2+^; 4. Cu_2_(*Eph*)_2_^2+^; 5. Cu(*Eph*)_2_^0^_(aq)_; 6. Cu(*Eph*)_2_OH^-^.


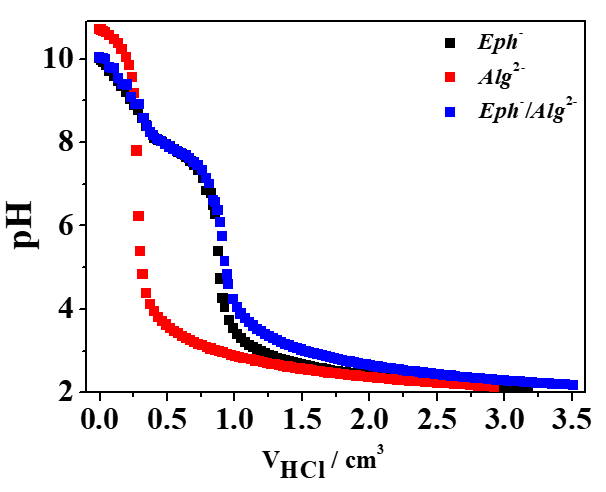


Figure S3. Comparison among the titration curves recorded for *Eph^-^*, *Alg^2-^* and *Eph^-^/Alg^2-^* systems at *Ī* = 0.146 mol L^-1^ in NaCl_(aq)_, *T* = 298.15K, *c_Eph_*_-_ = 3.00 mmol dm^-3^, *c_Alg_*_2_- = 1.50 mmol dm^-3^, *c*_NaOH_ = 1.18 mmol dm^-3^.

| ***^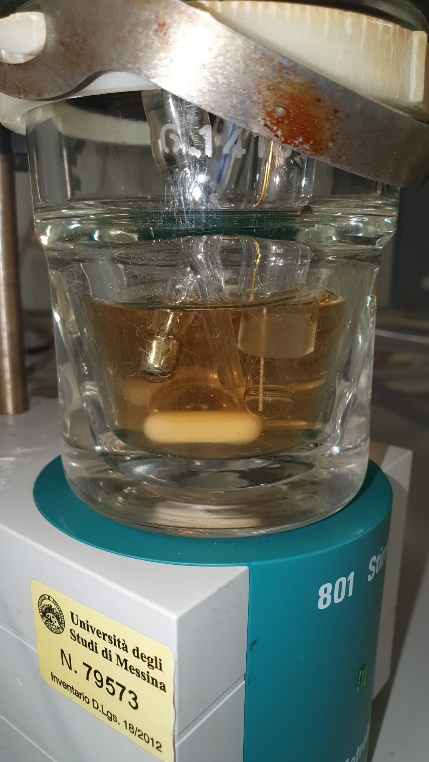^*** | ***^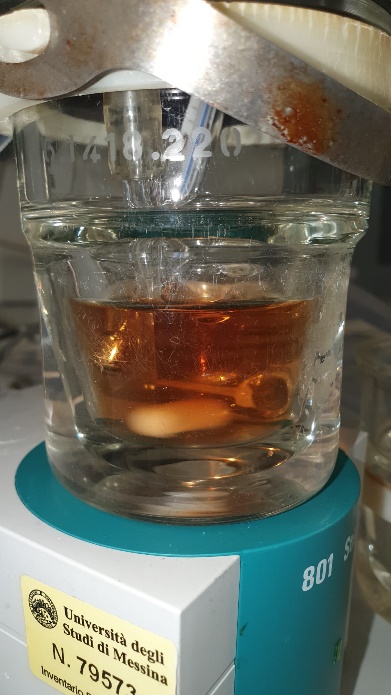^*** | 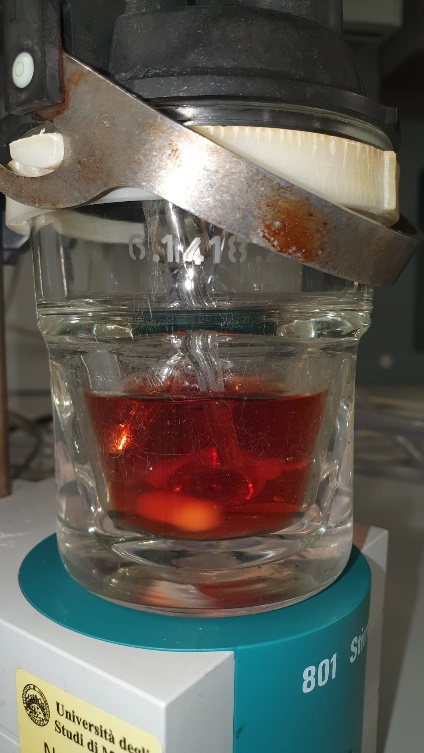 |
| --- | --- | --- |
| a) | b) | c) |

Figure S4. Colour variation of a Cu^2+^/*Eph*^-^/*Alg^2-^* measurement solution along the pH range of investigation.


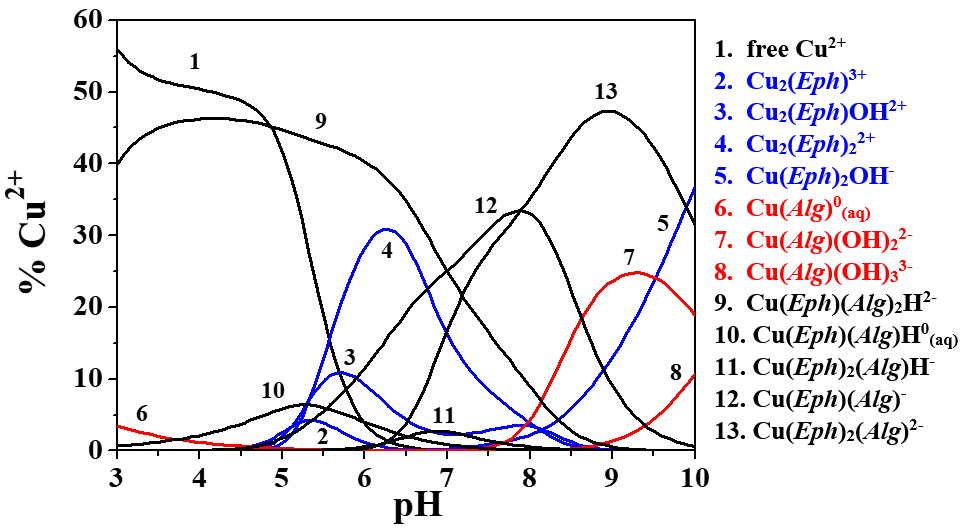


Figure S5. Distribution diagram of ternary Cu^2+^/*Eph*^-^*/Alg^2-^* system at *I* = 0.149 mol dm^-3^ in NaCl_(aq)_, *T* = 298.15K, *c*_Cu2+_ = 1.05 mol dm^-3^, *c_Eph_*_-_ = 1.40 mmol dm^-3^, *c_Alg_*_2-_ = 0.98 mmol dm^-3^.


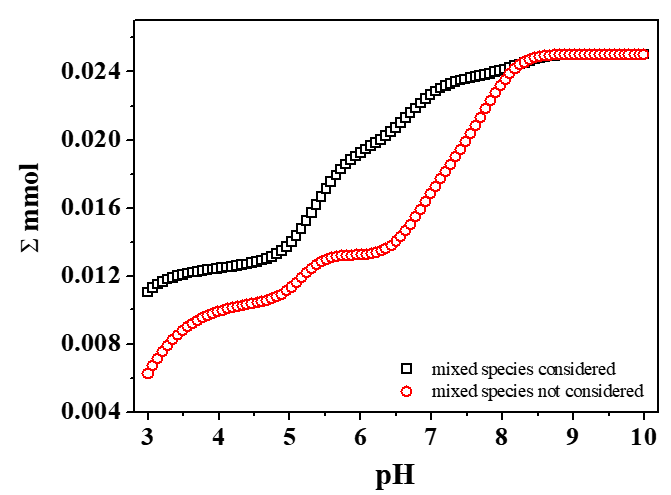


Figure S6. Comparison between the sum of mmoles for the Cu^2+^/*Eph*^-^/*Alg^2-^* complexes formation, considering and neglecting the ternary mixed species.


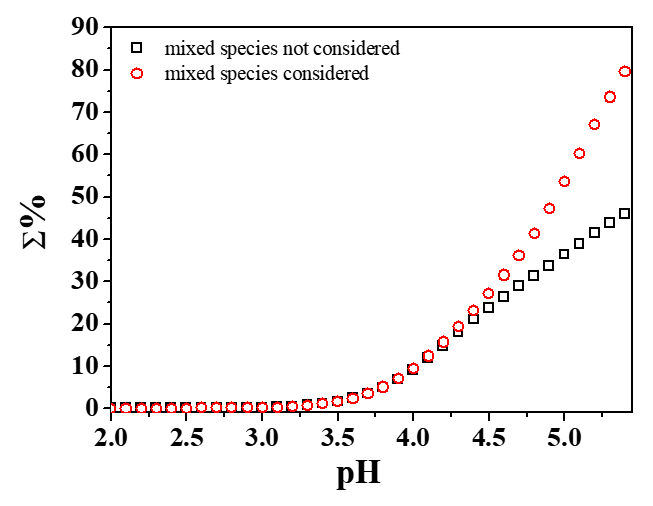


Figure S7. Comparison between the sum of the metals/ligand complexes formation percentages, considering and neglecting the ternary mixed species for the Cu^2+^/UO_2_^2+^/*Eph*^-^ species.

| 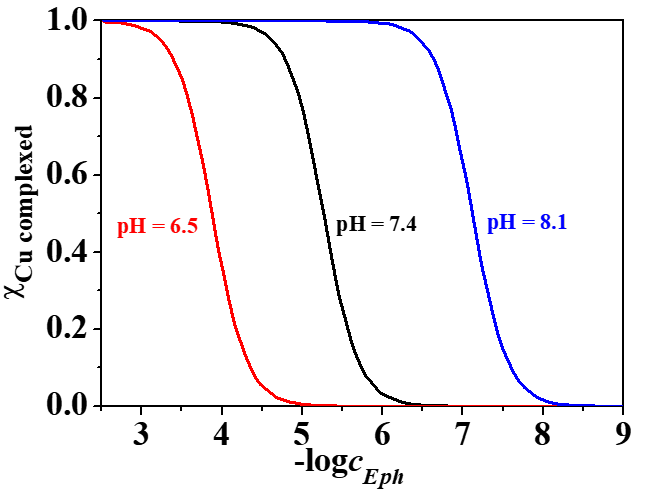 | 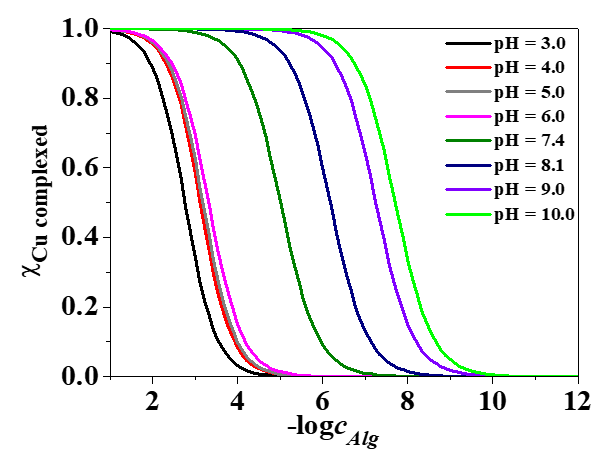 |
| --- | --- |
| *I* = 0.15 mol dm^-3^ | *I* = 0.15 mol dm^-3^ |
| a) | b) |
| ***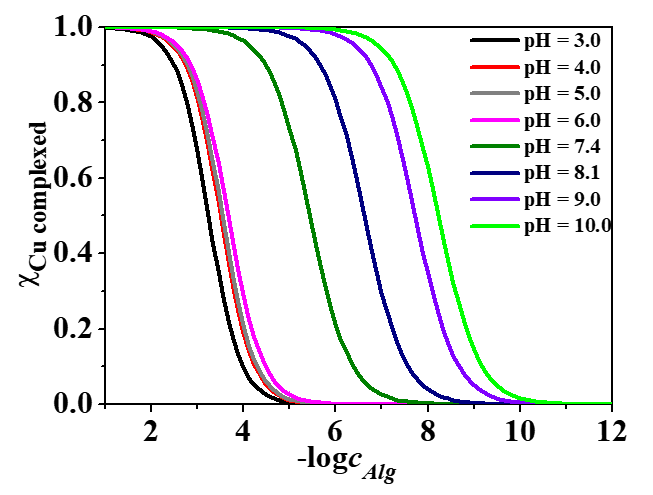***  *I* = 1.00 mol dm^-3^  c) | |

Figure S8. Sequestration diagrams of adrenaline (a) and alginate (b, c) towards Cu^2+^ at different pHs and ionic strengths (only for Alg^2-^) and *T* = 298.15K.

Figure S9 trend of pL_0.5_ vs pH for the Cu^2+^/*Alg*^2-^ system at different ionic strengths and *T* = 298.15K.
